# Integrative Analysis of *Diphasiastrum digitatum* Holub: Unveiling Genetic Variation and Ecological Adaptations for Sustainable Ecosystem Management

**DOI:** 10.1002/ece3.71079

**Published:** 2025-03-13

**Authors:** Marcin Nowicki, Logan C. Houston, Sarah L. Boggess, Matthew L. Huff, Margaret E. Staton, Robert N. Trigiano

**Affiliations:** ^1^ Entomology and Plant Pathology University of Tennessee Knoxville Tennessee USA; ^2^ Department of Regenerative Medicine Medical University of South Carolina Charleston South Carolina USA

**Keywords:** ecological niche modeling, evolutionary dynamics, fan clubmoss, phylogeography, Southern ground cedar, species management

## Abstract

Understanding the diversity and ecological evolutionary history of plant species is crucial for addressing the current biodiversity crisis and comprehending the processes by which organisms fill ecological and geographic spaces. In this study, we present a comprehensive analysis of the diversity and evolutionary history of *Diphasiastrum digitatum* Holub from the lycophyte lineage of plants, using microsatellite genotyping data and biogeographic analyses. Based on the available transcriptome assembly, we generated numerous markers and utilized 13 robust microsatellite markers to genotype a collection of 402 specimens from the Eastern US (VT; VA; NC; TN). In accordance with the accepted phylogeny, cross‐amplification tests demonstrated a closer relationship between *D. digitatum* and *Diphasiastrum* spp. compared with *Lycopodium* spp. Furthermore, the population genetics analyses identified two genetic clusters within the *D. digitatum* collection and suggested ongoing divergence and expansion. Isolation‐by‐distance analysis indicated that geographic distance had a minimal effect on differentiation, whereas environmental variables related to water regime were strongly associated with the genetic variance. Ecological niche modeling showed a post‐Last Glacial Maximum expansion of *D. digitatum* from southern refugia, corroborating a similar evolutionary scenario based on our microsatellite data. Overall, this study provides valuable insights into the evolutionary history of clubmosses and highlights the migration events and the environmental factors that shaped their current distribution.

## Graphical abstract

We convergently derived the evolutionary scenario for a basal lycopod species, *Diphasiastrum digitatum* , from genotyping data and ecological modeling. This information can better inform decisions about conservation efforts for this and related valuable lycopods.

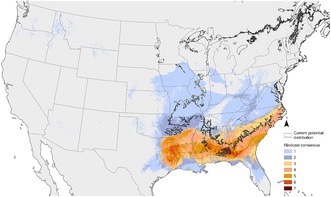

## 1 Introduction

The global impact of anthropogenic environmental changes, including habitat destruction and climate change, has led to increasing biodiversity loss, species extinctions, and alterations in ecosystem functioning. To inform downstream conservation efforts required for mitigating the loss of biodiversity, research is needed to understand the specific factors that influence population genetic diversity and species dynamics. Integrative approaches that combine molecular tools, evolutionary analyses, and ecological modeling have proved crucial for gaining a comprehensive understanding of species dynamics and informing conservation strategies (Cadotte et al. 2008).

Intraspecific genetic diversity plays a central role in shaping species interactions, evolutionary processes, and ecosystem functioning. A strong positive correlation between intraspecific genetic diversity and species richness was shown in terrestrial mammals, which indicates its significance in maintaining species richness in the face of environmental changes (Theodoridis et al. 2020). Tropical regions have experienced relatively stable climates over geological timescales, which have allowed the species to maintain large and stable populations and to reduce the likelihood of population bottlenecks and genetic drift, thereby preserving higher genetic diversity (Fonseca et al. 2023). Higher species richness in tropical regions often correlates with higher intraspecific genetic diversity because larger and more interconnected populations can maintain greater genetic diversity (Lawrence and Fraser 2020). The higher genetic diversity in tropical regions is also a result of various evolutionary processes, such as higher mutation rates, more frequent gene flow, and stronger selection pressures, which collectively contribute to the maintenance and generation of relatively higher genetic diversity (Adams and Hadly 2013). In contrast, glaciations caused notable range shifts and population size changes in temperate and polar regions. Species in these regions were often forced into isolated refugia, leading to population bottlenecks and reduced genetic diversity (Fonseca et al. 2023).

Compared to animals, genetic diversity in plants is generally lower and does not exhibit a consistent latitudinal pattern (De Kort et al. 2021). This discrepancy can be attributed to differences in life history traits, reproductive strategies, and dispersal mechanisms between plants and animals. For example, many plants can reproduce asexually or through self‐fertilization, which may reduce genetic diversity. Additionally, plant populations may be more influenced by local environmental conditions and less by historical climatic events compared to animals (Fonseca et al. 2023; Lawrence and Fraser 2020). Among plant communities, the relationship between biodiversity and ecosystem function, particularly the variation in their biomass, can be better explained by analyses of evolutionary relationships among species within a community compared to other diversity measures such as species richness or intraspecific diversity (Cadotte et al. 2008; Freschet et al. 2015; Luo et al. 2020; Shao et al. 2019). Such integrative approaches have helped discriminate the phylogeny and evolutionary history of several vascular plant lineages and provided insights into species dynamics and ecological interactions (Soltis and Soltis 1989; Wikström 2001; Su et al. 2021).

Isolation‐by‐Distance (IBD) and Isolation‐by‐Environment (IBE) are key concepts in understanding the evolutionary mechanisms that shape population‐level genetic differences. IBD refers to the increase in genetic differentiation between populations with increasing geographic distance, primarily due to limited dispersal and gene flow (Wright 1943). This pattern is expected in species with restricted dispersal capabilities, where gene flow decreases with distance, thus leading to greater genetic differentiation among distant populations. IBE occurs when genetic differentiation is more strongly associated with environmental differences than with geographic distance (Wang and Bradburd 2014). This pattern suggests that local adaptation to different environmental conditions drives genetic divergence, even among geographically proximate populations. IBE is particularly relevant in heterogeneous landscapes where environmental gradients can create strong selective pressures, leading to adaptive genetic differentiation.

Several evolutionary mechanisms contribute to population‐level genetic differences, including natural selection, genetic drift, gene flow, and mutation. Natural selection acts on genetic variation within populations, favoring alleles that confer a selective advantage in a given environment. This process can lead to local adaptation and increased genetic differentiation among populations experiencing different environmental conditions (Kawecki and Ebert 2004). Genetic drift, the random fluctuation of allele frequencies, can also contribute to genetic differentiation, particularly in small populations where chance events can have larger impacts. Gene flow, the movement of alleles between populations, counteracts genetic drift and selection by homogenizing allele frequencies across populations. Under limited gene flow, populations can diverge genetically due to the combined effects of drift and selection (Slatkin 1987). Mutation introduces new genetic variation into populations, providing the raw material for evolution. The interaction of these mechanisms shapes the genetic structure of populations and their ability to adapt to changing environments.

*Diphasiastrum digitatum* Holub (formerly *Lycopodium digitatum* Dill. ex A. Braun) is a member of the lycophyte lineage of plants, commonly known as fan clubmoss or Southern ground cedar. It is ecologically important in terrestrial ecosystems as a late successional species due to the rapid vegetative expansion of its rhizomes (Callaghan 1980; Singleton et al. 2001; Hornbeck et al. 2002; Aagaard et al. 2009; Klein 2012; Rimgailė‐Voicik 2017). The species' life cycle begins with the release of airborne spores from above‐ground sporophytes, which can grow up to 15 cm tall (Figure S1; Singleton et al. 2001; Klein 2012). These spores are lightweight and can be carried over long distances by air currents, facilitating gene flow between distant populations. These spores disperse and find suitable conditions to develop into long‐lasting, soil‐buried gametophytes. To be successful, the gametophytes require fungal symbionts for growth and development (Kuprina et al. 2025; Winther and Friedman 2008; Horn et al. 2013; Pawłowska et al. 2014). Over time, the gametophytes give rise to new sporophytes, completing the cycle. The long‐lived subterranean gametophytes of *D. digitatum* are noteworthy, as they can persist in the soil for extended periods, potentially decades, before developing into sporophytes. This longevity allows the species to survive adverse environmental conditions and take advantage of favorable conditions when they arise (Wagner 1990; Gilman 2001; Whittier 2003; Rimgailė‐Voicik 2017). The requirement for fungal symbionts further underscores the ecological complexity and interdependence within their habitats. The potential for long‐distance spore dispersal is another critical feature of *D. digitatum* . The lightweight spores can be dispersed by wind over considerable distances, which facilitates gene flow and reduces genetic differentiation among populations. This dispersal mechanism is crucial for maintaining genetic diversity and enabling the colonization of new habitats. It also implies that *D. digitatum* can respond to environmental changes by shifting its range, which is particularly relevant in the context of climate change.

*Diphasiastrum digitatum* (2n = 2× = 46; 1C: 2.79 pg. or 2729 Mbp; Wang et al. 2005; Bainard et al. 2011) is native to eastern North America, where its range overlaps with closely related *D. complanatum* and *D. tristachyum* (Klein 2012) to varying degrees. Whereas the geographic ranges of *D. digitatum* and *D. tristachyum* overlap considerably in eastern North America, the range of *D. complanatum* stretches comparably much more into the North (Rumsey et al. 2021; Lin et al. 2024). The ecological niches of the three species differ as well. *Diphasiastrum digitatum* is commonly found in dry to mesic, acidic coniferous forests and disturbed areas, and forms dense monocultures that dominate the forest floor (Rumsey et al. 2021). Comparably, *D. complanatum* prefers cooler, moist habitats in shaded, coniferous forests, but is more scattered than *D. digitatum* . Finally, despite major geographical range overlapping with *D. digitatum* , *D. tristachyum* tends to specifically occupy more sandy soils and open woodlands, where it can tolerate more sunlight and is less competitive in dense forest environments (Rumsey et al. 2021; Lin et al. 2024).

Currently observed ranges of species distribution may help infer its evolutionary past and evaluate scenarios for potential impacts under the impending climate change. The concept of a southern refugium for *Diphasiastrum digitatum* is supported by biogeographic and paleoclimatic evidence. During the Last Glacial Maximum (LGM), approximately 21,000 years ago, much of North America was covered by ice sheets, which forced many species to retreat to more southerly locations where the climate remained suitable for their survival. These refugia served as safe havens where species could persist through glacial periods and later expand northward as the climate warmed. For *D. digitatum* , the southern Appalachian region likely served as a major refugium. This area is known for its high biodiversity and has been identified as a refugium for various plant and animal species during past climatic fluctuations (Ony et al. 2021). The relatively stable climate and diverse habitats in this region provided suitable conditions for the survival and persistence of *D. digitatum* populations.

The ecological importance, genetic diversity, population structure, and evolutionary history of *Diphasiastrum digitatum* remain underexplored. To address this knowledge gap, we developed and utilized a set of microsatellite markers to genotype an extensive collection of *D. digitatum* specimens and examine population‐level genetic patterns. Specifically, our objectives included investigating the genetic structure, demographic history, and environmental factors influencing the distribution of *D. digitatum* populations. Based on prior macroscopic observations, we hypothesized that *D. digitatum* exhibits population structuring influenced by environmental factors, such as water‐ (Ricklefs 2006; Shao et al. 2019) or temperature‐related variables (Chevalier et al. 2024; Zurell et al. 2024). This hypothesis was driven by the observations of the combination of rhizome severing and nutrient addition having the most pronounced negative vigor impact (Railing and McCarthy 2000). It was also supported by non‐Fibonacci leaf arrangement suggestive of unique developmental processes in *D. digitatum* (Singleton et al. 2001). Observations of monoculture formation and ecological resilience (Hornbeck et al. 2002; Vogel et al. 2011) and distinct ecological preferences (Klein 2012) lent further support to this overarching hypothesis. We aimed to enhance our understanding of the genetic diversity, population dynamics, and ecological niche of *D. digitatum* in our collection that focused on the populations from Virginia Creeper National Recreation Trail in Virginia, USA (‘VCT’) sampled most intensively to analyze differences in population genetic structure along the altitudinal gradient of the trail. Other sites in North Carolina, Vermont, and Highland Rim, Tennessee were sampled to provide additional context (‘NC’, ‘VT’, ‘HR_TN’, respectively), to attempt to disentangle the relationships between increasing elevation and increasing latitude as spatial factors that shape the expression of coarse‐resolution climatic variables. Specifically, we explored the relationship between genetic variation across a gradient of both elevation and latitude, with the broad‐scale variation among the four regional sites (‘VCT’, ‘VT’, ‘TN’, ‘NC’) contrasted with the fine‐scale variation among subpopulations at the ‘VCT’ site. We expected to find varying levels of genetic diversity across different sites. Given the species' wide distribution and the potential for local adaptation, we hypothesized that populations in different geographic regions would exhibit distinct genetic profiles. In populations located in regions with stable environmental conditions and high habitat connectivity (‘VCT’), we expected to find higher gene flow, leading to greater genetic diversity (Fonseca et al. 2023; Lawrence and Fraser 2020).

We also explored the patterns of historical demographic and ecological range shifts. We hypothesized that historical demographic events, such as post‐glacial expansions and population bottlenecks, have shaped the current genetic structure of *Diphasiastrum digitatum* populations. The concept of refugia is particularly relevant for this study system, as it can provide insights into how *D. digitatum* populations have persisted through historical climatic changes and how these refugia have influenced current genetic patterns. This knowledge will inform conservation and management efforts for this taxon and related species, thereby highlighting the importance of integrated approaches in safeguarding biodiversity in a changing world (Klein 2012; Hanušová et al. 2014; Schnittler et al. 2019). Although *D. digitatum* is not currently listed as endangered, it is recognized as vulnerable in some states, with a few regions considering it critically imperiled (https://explorer.natureserve.org/Taxon/ELEMENT_GLOBAL.2.138158/Lycopodium_digitatum). Globally, *D. digitatum* holds a status of G5, indicating widespread abundance and security. By providing insights into the ecological significance of *D. digitatum* and its implications for conservation and management strategies, our study lays the groundwork for proactive conservation efforts that are linked to the long‐term health of forest ecosystems.

## 2 Materials and Methods

### 2.1 Plant Materials

**FIGURE 1:**
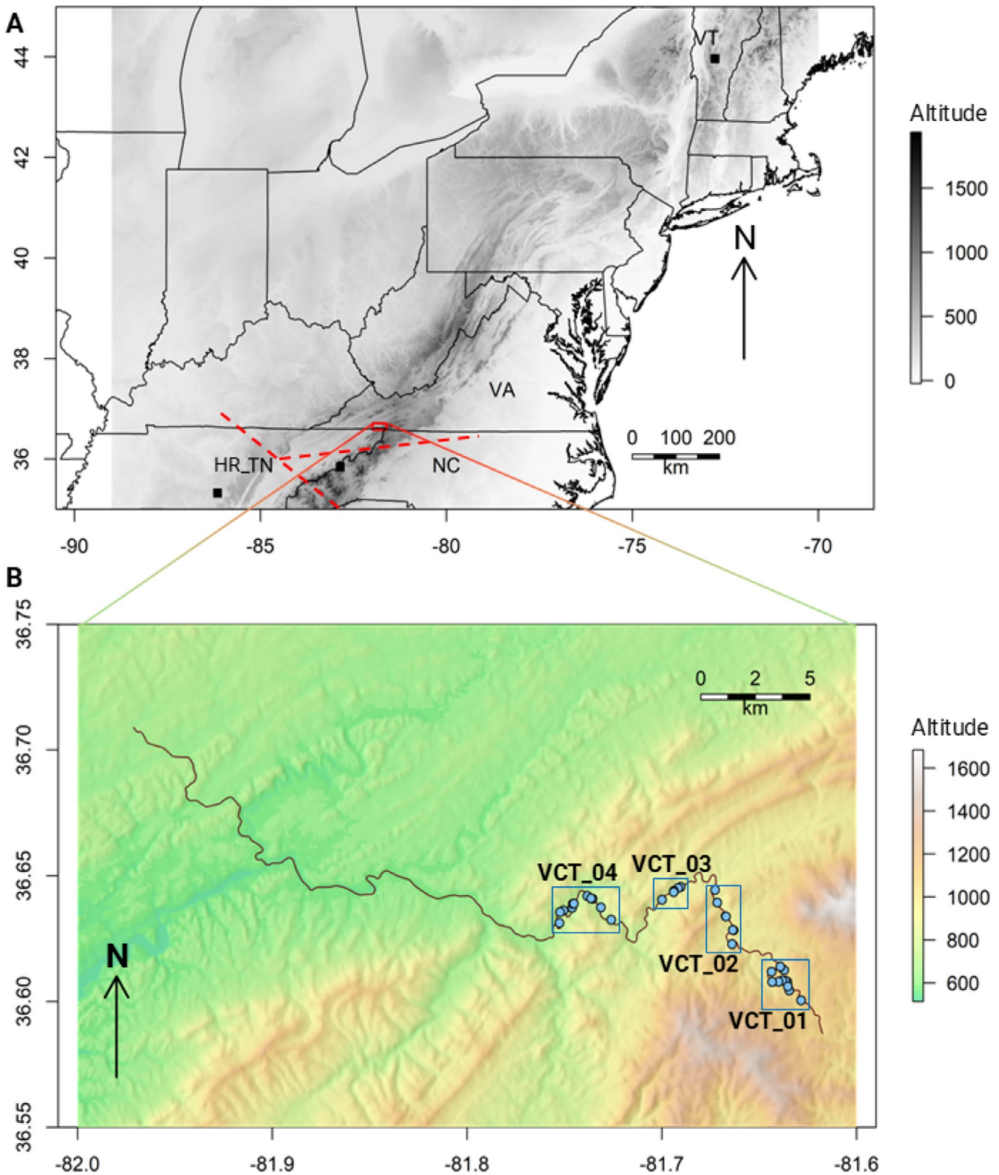
*Diphasiastrum digitatum* collections. (A) Eastern US region with black squares (■) denoting collection sites in three states (NC; TN; VT) and the red rectangle delimiting the Virginia Creeper Trail (VA), shown with an inset map in (B). The dotted red line in the top panel delimits separation of populations and is derived from the Barrier analysis. (B) Inset from A: Collection sites (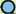) along the Virginia Creeper Trail (dark purple line), grouped into four numbered populations (‘VCT_01’ to ‘VCT_04’). Maps were drawn to scales, respectively, and the direction for North (N) is shown with arrows. Legends on the right code the colors for altitude (m.a.s.l.). *x*‐axes: Longitude; *y*‐axes: Latitude.

Several species of *Diphasiastrum* were sampled in the eastern US. Thirty‐two collection sites (or ‘plots’) of *D. digitatum* (Figure S1) were sampled in May and June of 2018 along the Virginia Creeper National Recreation Trail in Virginia, USA (‘VCT’; *n* = 353 individuals; Figures 1 and S1) along with locations in North Carolina (‘NC’; *n* = 15 individuals), Vermont (‘VT’; *n* = 22 individuals), and Highland Rim, Tennessee (‘HR_TN’; *n* = 12 individuals) (Figure 1B; Tables 1, S1). Related permit 2720 (2018) was issued by the Forest Supervisor at USDA‐FS George Washington and Jefferson National Forest in southwest Virginia. The moderate climate with warmer summers and milder winters at upper elevations in ‘VCT’ region results in longer growing seasons, especially compared with ‘VT’ region.

**TABLE 1:** Indices of diversity in populations of *Diphasiastrum digitatum* .

Population	Sample size	% missing^a^	NA/pop	NA	NA_e_	AR	H_e_	H_o_	F_i_	Pa	$\bar{r}$_*d*_	F_null_	HET_exc_	HET_def_
VCT_01	124	2.2 (1.8%)	113	8.69	3.90	5.50	0.62	0.41	0.34****,^ns^	9	0.06****	0.12	1	0.0000
VCT_02	62	0.0 (0.0%)	100	7.69	4.26	5.80	0.70	0.43	0.39****	0	0.11****	0.15	1	0.0000
VCT_03	49	0.0 (0.0%)	115	8.85	4.97	6.37	0.71	0.44	0.38****	10	0.13****	0.15	1	0.0008
VCT_04	108	1.9 (1.8%)	131	10.08	4.90	6.46	0.73	0.39	0.46****	11	0.05****	0.19	1	0.0000
NC	15	0.0 (0.0%)	55	4.23	2.87	4.08	0.54	0.39	0.30****	1	0.19****	0.17	1	0.0011
VT	22	0.2 (0.7%)	102	7.85	5.03	6.55	0.68	0.37	0.46****	4	0.01^ns^	0.17	1	0.0013
HR_TN	12	0.0 (0.0%)	56	4.31	3.13	4.31	0.56	0.37	0.35****	0	0.10****	0.16	1	0.0284
Overall	392	4.3 (1.1%)	672	12.85	5.16	6.64	0.71	0.41	0.43****	35	0.04****	0.16	1	0.0045

^a^ The following indices are presented: Number of missing genotypes (and % per population); Number of alleles per population (NA/pop); Number of alleles (NA); Effective number of alleles (NA_e_; (Nielsen et al. 2003)); Allelic richness (AR) rarefied to 24 gene copies; Gene diversity corrected for sample size (H_e_; (Nei 1978)); Observed heterozygosity (H_o_); Individual inbreeding coefficient (F_i_); Number of private alleles (P_a_); Standardized index of association ($\bar{r}$_*d*_); frequency of null alleles (F_null_; Van Oosterhout et al. 2004); Significance of alternative hypothesis for heterozygote excess (HET_exc_) or deficiency (HET_def_; Van Oosterhout et al. 2004).

**** Significance levels are denoted, wherever applicable: *p* < 0.0001.

^ns^ Significance levels are denoted, wherever applicable: ns, not significant.

Based on the geographical proximity and the separation from other ‘VCT’ collection sites, the Virginia Creeper Trail specimens of *D. digitatum* were grouped into four subpopulations (‘VCT_01’, ‘VCT_02’, ‘VCT_03’, ‘VCT_04’; Figure 1; Tables 1, S1); the other three collection sites (‘NC’; ‘VT’; ‘HR_TN’) were all considered distinct subpopulations. At each collection site, the coordinate location was recorded by GPS (± 1 m) and later annotated for altitude using the Elevation Finder tool (https://www.freemaptools.com/elevation‐finder.htm). Samples within each collection site were taken at least 5 m apart to ensure they were not the same specimen. A group of *Lycopodium* spp. from the Virginia Creeper Trail (*n* = 10 individuals; presumably *L. clavatum* L. as the only species of *Lycopodium* s.s. present in Virginia (W. Testo, *pers.comm*.)), along with individual samples of *D. complanatum* × *sieitatum* (=*D. × verecumdum* (L.) Holub; designated by A.V. Gilman), *D. tristachyum* ((Pursh) Holub), and *D*. × *sabinifolium* ((Willd.) Holub; designated by A.V. Gilman) collected in Vermont, were included to test the cross‐amplification of the molecular simple sequence repeat markers (SSRs) used.

### 2.2 DNA Extraction

Samples of plant stems were homogenized 3 times using a BeadMill 24 (ThermoFisher Scientific, Waltham, MA, USA) at a setting of S (speed) = 6.0 m/s and T (time) = 30 s. Samples were frozen in liquid nitrogen between each homogenization round. DNA was extracted using the Omega Plant DS kit (Omega Bio‐tek Inc., Norcross, GA) following the manufacturer's protocol, with the following exceptions: heating the elution buffer to 65°C before use and eluting with 50 μL two times. DNA was quantified using a Nanodrop 1000 spectrophotometer (ThermoFisher Scientific, Waltham, MA).

### 2.3 Primer Screening and Optimization

Primers for SSRs were developed based on an RNASeq dataset of *Diphasiastrum digitatum* (One Thousand Plant Transcriptomes Initiative 2019; NCBI short‐read archive ERR2040872). The RNASeq data was assembled using SOAPdenovo‐Trans (Xie et al. 2014) and consisted of 69,669 individual transcripts. The transcriptome was masked of low‐complexity regions with DustMasker (Morgulis et al. 2006). An in‐house software pipeline (https://github.com/statonlab/Finding‐SSRs) was utilized to search that dataset for SSR sequences with 8–30 dinucleotide repeats, 7–20 trinucleotide repeats, or 6–20 tetranucleotide repeats. Primer3 (Untergasser et al. 2012) was used to construct primers. Fifty primer pairs of di‐ (*n* = 31) and trinucleotide repeat SSRs (*n* = 19) were randomly selected and synthesized (Integrated DNA Technologies Inc., Coralville, IA). An initial optimization and screening of these 50 SSR markers was completed using 6 randomly selected DNA samples of *D. digitatum* collected from the Virginia Creeper Trail. Based on expected PCR product sizes, polymorphism of the amplified PCR products, and rates of successful amplification, 13 primer pairs were selected for the remainder of the study (Table S2).

PCR amplifications were completed in 10 μL reactions consisting of the following: 4 ng of genomic DNA, 0.5 μM each of forward and reverse primers, 5 μL of 2× AccuStart II PCR SuperMix (QuantaBio, Durham, NC), and 0.5 μL of DMSO. A positive control (reaction with a DNA sample that worked across all loci during primer screening) and a negative control (PCR mixture with no DNA added) were included in each experimental PCR 96‐well plate to ensure the validity of the data. Amplifications were completed under the following thermal profile: 94°C for 3 min, 15 cycles of touch‐down: 94°C for 40 s, 63°C–0.6°C/cycle for 15 s, 72°C for 30 s, and 20 cycles of 94°C for 40 s, 57°C for 40 s, 72°C for 30 s, with a final extension of 72°C for 4 min. Amplified PCR fragments were visualized using a QIAxcel Advanced Capillary Electrophoresis system (Qiagen, Germantown, MD), aligned using an internal 15/600 bp alignment marker (Qiagen), and sized based on the 25–500 bp size marker (Qiagen). Fragments were analyzed using QIAxcel ScreenGel v.1.6.0.10 (Qiagen). All reactions that failed to amplify were subjected to two additional attempts before being scored as missing data. If a sample had missing data for more than two SSRs, it was discarded from the dataset.

This resulted in 402 genotyped samples of *Diphasiastrum digitatum* from 7 subpopulations retained for the subsequent data analyses. The FlexiBin MS Excel macro (Amos et al. 2007) was used to bin the raw allele sizes into statistically similar allelic classes. Due to the constraints of the Qiaxcel system (Qiagen), manual binning was completed to group allele categories together to ensure 4 bp or greater spacing between each allelic class for each analyzed locus. The binned dataset was transformed to repeat number using the motif size information in PGDSpider version 2.1.1.5 (Lischer and Excoffier 2012) and clone‐corrected at the subpopulation level using the package *poppr* v2.1.5 (Kamvar et al. 2014) in R v3.6.1 (R Development Core Team 2023). The resulting dataset consisted of 392 unique multi‐locus genotypes (MLGs) of *D. digitatum* that were subjected to all subsequent analyses. Genomic locations of the SSRs selected based on their interesting characteristics (see Results and Discussion) were assessed by retrieving the contigs in which the SSRs were located and using the NCBI Blast (Altschul et al. 1990) reqseq‐rna database within Lycopodiales on such retrieved contigs.

### 2.4 Hierarchical Population Statistics

Basic population‐ and locus‐wise statistics were computed in R using *poppr*, *hierfstat* v0.5‐10 (Paradis 2010), *pegas* v1.0‐1 (Paradis 2010), *adegenet* v2.1.5 (Jombart 2008), and *lattice* v0.20‐45 (Sarkar, Sarkar et al. 2015). These included the following: percentage of missing data; number of alleles (NA) and effective alleles (NA_e_; Nielsen et al. 2003); allelic richness (AR); heterozygosity expected under Hardy‐Weinber equilibrium (H_e_; Nei 1978); observed heterozygosity; Jost's D index of differentiation (Jost 2009; Jost et al. 2018); individual inbreeding coefficient (F_i_); the R‐indices of inbreeding within samples/differentiation among samples (R_is_; R_st_; Slatkin 1995; Michalakis and Excoffier 1996; Hardy and Vekemans 2002); and the standardized index of association ($\bar{r}$_*d*_). Private alleles at subpopulation level (P_a_) and gene flow index (N_m_) were computed using the MS Excel macro GenAlEx v6.5.1 (Peakall and Smouse 2012). SPAGeDI v1.5 (Hardy and Vekemans 2002, 2015) was used to calculate the significance for the basic hierarchical statistics based on 1999 dataset permutations and to assess the presence of phylogenetic signals among and within populations. Null alleles frequency, heterozygote excess, and heterozygote deficiency were assessed using micro‐checker v2.2.3 (Van Oosterhout et al. 2004), specifically at 1000 permutations of dataset, 95% confidence interval, and Brookfield's 1996 method (Brookfield 1996). Null allele frequencies below 0.2 are often considered acceptable in population genetic studies (Li et al. 2017; Jahnke et al. 2022). Frequencies above that threshold might indicate potential issues with the primers or the quality of the DNA samples, which could necessitate further investigation or adjustments.

Partitioning of the molecular variance was assessed using the analysis of molecular variance (AMOVA) implemented in *poppr*, and the significance of these results was tested using 999 dataset permutations. To examine the levels of selfing, AMOVA was conducted with and without the within‐individuals partitioning, dubbed as 3‐tier or 2‐tier, respectively.

### 2.5 Population Structure and Ecological Niche Modeling

To test the isolation‐by‐distance (IBD), a Mantel test implemented in R packages *MASS* v7.3‐54 (Ripley et al. 2013), *ade4* v1.7‐18 (Dray and Dufour 2007), and *vegan* v2.5‐7 (Oksanen et al. 2013, 2019) was employed. A partial‐Mantel test to analyze the influence of altitude on the IBD was completed using the same software. Environmental data were downloaded from WorldClim (Fick and Hijmans 2017) at a resolution of 30 arc sec, equivalent to approximately 0.876 km squared at the latitude of the Virginia Creeper Trail. A matrix of the ecological distances created using the dist() function in R was then related to the geographical distances to assess isolation‐by‐environment (IBE) in a way analogous to IBD. To identify the WorldClim environmental variables associated with the inferred genetic clusters at the level of 35 collection sites, we used the *rpart* package v4.1.16 (Therneau et al. 2015) in R. The x2y R script (Ramakrishnan 2021) computes the percentage of observations in the dataset that were used to assess each given variable pair's metrics and the corresponding confidence interval based on 1000 bootstraps. The Redundancy Analysis (RDA) of the WorldClim variables deemed significantly associated (see above) and longitude, latitude, altitude, and the inferred genetic clusters was performed in R, using packages *vegan*, ‘sklearn.decomposition’ module (Pedregosa et al. 2011) in Python v0.24.2, *ggplot2* v3.3.5 (Wickham 2016), *matplotlib* v3.4.3 (Hunter 2007), *viridis* v.0.6.1 (Garnier 2018), *seaborn* v.0.11.2 (Waskom et al. 2020).

To assess the presence of population structure within the genotyped dataset, the Bayesian approach implemented in structure v2.3.4 (Pritchard et al. 2000) was employed. The analysis was run in the admixture mode and included 500,000 burn‐in iterations followed by 500,000 steps of sampling in 20 parallel Monte Carlo Markov Chains across the range of inferred genetic clusters *K* = 1–10. The results were analyzed for the optimum K using the Evanno approach (Evanno et al. 2005), implemented in the PopHelper webapp v1.0.10 (Francis 2017), followed by similar visualizations for other, less‐supported values of K. Subsequently, ObStruct v1.0 (Gayevskiy et al. 2014) was used to assess the relationship between the pre‐attributed subpopulations and the genetic clusters inferred using structure at the optimum K. The ad‐hoc parameter *R* ^2^ ranges from 0, denoting recent divergence or strong admixture of subpopulations, to 1, denoting complete subpopulation divergence or strong structure. Furthermore, the same program was used to investigate changes in *R* ^2^ when iteratively removing one of the pre‐attributed subpopulations or the inferred clusters to estimate their impact on the overall score.

The population structure of the genotyped dataset was assessed using Discriminant Analyses of the Principal Components (DAPC), an ordination technique implemented in *adegenet*. This analysis was optimized and cross‐checked with 100 permutations across the Principal Components (PCs) used for projection ranging from 2 to 152 (total number of detected alleles). The matrix of genetic distances among subpopulations generated using the *poppr* function ‘diss.dist()’, equal to Prevosti distance (Prevosti 1974), was optimized for the clustering algorithm using the package *adegenet*. The matrix of distances served to generate a tree using the package *phangorn* v2.8.1 (Schliep 2011) in R based on the most‐parsimonious tree, bootstrapped 999 times, and visualized in R as an unrooted neighbor‐joining tree.

The presence of genetic barriers among the population was assessed using Barrier v.2.2 (Manni et al. 2004), utilizing the algorithm of maximum difference (Monmonier 1973). The matrix of pairwise Prevosti distances (Prevosti 1974) between subpopulations was compared with the respective geographical coordinates of their origins using 100 bootstraps. The barriers with the highest relative values were reported.

### 2.6 Population Demography and Evolutionary History

The evidence of recent genetic bottleneck was examined using the program Bottleneck v1.2.02 (Cornuet and Luikart 1996; Piry et al. 1999), to assess the assumed hypothesis that all loci fit mutation‐drift equilibrium under relevant mutation models. This analysis computed 10,000 permutations under the stepwise mutation model (S.M.M.) and the two‐phase model (T.P.M.; with 95% S.M.M. and 5% multiple‐step mutations and a variance among multiple steps of approximately 12). Analyses of heterozygosity excess or deficiency included the sign test, the standardized differences test, and the Wilcoxon sign‐rank test. Based on the cumulative results of all three tests, a qualitative descriptor of allele frequency distribution informs the presence of a given subpopulation bottleneck (“shifted mode”) or lack thereof (“normal L‐shaped distribution”).

Analyses of the evolutionary history of *Diphasiastrum digitatum* in the eastern US used the program DIYABC v2.1.0 (Cornuet et al. 2010, 2014) implementing the approximate Bayesian approach. To calculate the initial parameter values, the entire dataset was considered one population, with all parameters under uniform distribution. The effective population size was set between 10 and 100,000 individuals, and mutation rates between 0.00001 and 0.001 mutations per locus per generation under complete S.M.M. and with no requirement for all individual loci to take the same value (shape = 2). DIYABC then generated 1000,000 pseudo‐observed datasets (PODs) under these constraints and calculated the following population statistics at the subpopulation level: mean number of alleles, mean genetic diversity, mean size variance, and mean Garza–Williamson's M. At the pairwise subpopulation level, the statistics calculated included: mean number of alleles, mean genic diversity, mean allele size variance, Fst, classification index, shared allele distance, and (dμ)^2^ distance (Cornuet et al. 2014). When these were projected using the implemented Principal Components Analysis, data from 1% of the PODs closest to the genotyped dataset were used to calculate the effective population size and the mutation rates.

The 95% confidence interval (95% CI) ranges of these parameter values (population sizes between 500 and 10,000; mutations rates same as above) were then used in the actual run, which compared 12 competing evolutionary scenarios over 3,000,000 PODs. In this analysis, four subpopulations from Virginia Creeper Trail (‘VCT_01’, ‘VCT_02’, ‘VCT_03’, ‘VCT_04’) were combined to substantially decrease the number of scenarios considered and the time of computation, as done in other studies (Ony et al. 2021; Sapkota et al. 2022). In all devised scenarios, the combined Virginia Creeper Trail population (‘VCT’) was considered an intermediary population due to its central geographic location relative to the other populations being studied. Logistic regression was used to identify the two best‐performing scenarios, which were compared in the final run of 2,000,000 PODs. For the best‐supported scenario, bias and accuracy were calculated, and the top two performing scenarios were used to analyze the confidence in scenario choice: the global prior and posterior errors as well as type I and type II scenario‐based (prior) errors.

### 2.7 Ecological Niche Modeling Using MaxEnt

Occurrence data of *Diphasiastrum digitatum* were downloaded from GBIF (https://www.gbif.org/occurrence/map?taxon_key=2688375&occurrence_status=present) and combined in a single file. The entire dataset was filtered according to the following steps. Any records with missing collection/observation year, latitude and longitude data, vague locality descriptions, or spatial uncertainty (GPS rounding) exceeding 1 km were excluded. The temporal range (1976–2015) of records was limited to adhere to the current climate regime. The most recent records (2016–2022) and the records from the samples in this study were used for additional model testing (omission error). Current bioclimatic variables, representing the climate normals for 1981–2010, were downloaded from the CHELSA database v2.1 (https://chelsa‐climate.org/downloads/). The same database included 7 Last Glacial Maximum (LGM) reconstructions with the same bioclim variables as for the current normals: CCSM4; CNRM‐CM‐5; GOALSg2; IPSLCM5ALR; MIROC‐ESM; MPIESMP; MROCGCM3. MaxEnt v3.4.0 (Phillips et al. 2004) models were calibrated with the current normals (baseline bioclim variables) and then individually projected to each of the 7 LGM reconstructions. This generated 7 possible LGM distributions of *D. digitatum* .

The current MaxEnt model was fit with occurrence records from 1976 to 2015 and all 19 bioclim variables as predictors. Bootstrapping with 100 replicates was used to evaluate the calibration/validation of models with subsets of the occurrence records. The MaxEnt model was also used to evaluate the contribution of each predictor variable to the model accuracy gain. Correlation analysis of the bioclim variables was used to check for collinearity and to further reduce the number of variables with correlation coefficients > 0.7. The final model for current species distribution used occurrence data and a subset of bioclim variables. It was then projected on 7 LGM reconstructions with a spatial extent beyond the training region of the current species distribution.

## 3 Results

### 3.1 SSR Development, Genotyping, and Cross‐Amplification

The assembled transcriptome of *Diphasiastrum digitatum* yielded 3609 sequences with at least one SSR, with a total of 3934 SSRs. After the removal of 168 compound SSRs, primers were constructed for 1134 SSRs, which included 568 di‐, 374 tri‐, and 192 tetra‐nucleotides (Figure S2). All unique di‐nucleotides and their redundant iterations were represented. Among the tri‐nucleotide motif groups, only [GCG]_n_/[GTA]_n_ and their redundant iterations were not detected. Among the tetra‐nucleotides, 96 unique motifs out of 236 were represented, equivalent to 25 redundant motifs present out of 33 redundant motif groups. The di‐nucleotide motifs were the most frequently found among those with primers (Figure S2). After the screening of 50 SSR primer pairs using 6 randomly selected DNA samples of *D. digitatum* , we retained 6 di‐ and 7 tri‐nucleotide SSRs (Tables 2, S2) to genotype the collection of *D. digitatum* and to test the cross‐amplification into related species.

**TABLE 2:** Indices of diversity in loci of 392 specimens of *Diphasiastrum digitatum* , calculated at data subdivision as presented in Table 1 (i.e., 7 subpopulations).

Locus	% missing^a^	NA	NA_e_	AR	H_e_	H_o_	R_is_	R_st_	Jost's D	N_m_	F_null_	HWE_exc_	HWE_def_
Lyco008	31 (7.9%)	11	5.43	7.14	0.82	0.32	0.67****	0.27****	0.59	0.94	0.27	1.00	0.0187
Lyco009	0 (0.0%)	3	1.11	1.92	0.10	0.00	1.00****	0.02^ns^	0.00	5.97	0.16	1.00	0.0085
Lyco015	11 (2.8%)	24	7.80	9.16	0.87	0.44	0.18*	0.05W	0.43	2.65	0.24	0.86	0.0000
Lyco016	1 (0.3%)	24	15.24	12.69	0.93	0.82	−0.14**	0.03****	0.55	3.10	0.06	0.74	0.0022
Lyco017	1 (0.3%)	8	2.06	3.79	0.51	0.11	0.27**,^ns^	0.05*	0.12	1.46	0.26	1.00	0.0011
Lyco020	0 (0.0%)	20	8.48	9.53	0.88	0.77	0.14**	0.18****	0.46	2.74	0.05	0.62	0.0188
Lyco022	2 (0.5%)	10	3.42	5.31	0.71	0.32	0.11^ns^	0.05**	0.30	1.32	0.23	1.00	0.0014
Lyco024	4 (1.0%)	11	3.60	5.86	0.72	0.16	0.62****	0.05****	0.35	1.55	0.32	1.00	0.0000
Lyco030	0 (0.0%)	15	4.02	6.82	0.75	0.47	−0.11^ns^	0.08****	0.25	2.53	0.16	0.84	0.0002
Lyco031	0 (0.0%)	9	3.74	6.57	0.73	0.52	0.09^ns^	0.03****	0.17	2.97	0.06	0.85	0.0005
Lyco035	6 (1.5%)	12	3.13	5.36	0.68	0.54	−0.13*	0.02*	0.05	7.12	0.08	0.85	0.0062
Lyco044	0 (0.0%)	8	3.51	5.59	0.71	0.27	0.24****	0.12****	0.21	2.51	0.24	0.96	0.0001
Lyco048	0 (0.0%)	12	5.55	6.57	0.82	0.56	0.22****	0.12****	0.50	1.15	0.14	0.85	0.0009
Multilocus average	4.3 (1.1%)	12.85	5.16	6.64	0.71	0.41	0.05^ns^	0.07****	0.24	2.77	0.17	0.89	0.0045

^a^ The following indices are presented: Number of missing genotypes (and % per population); Number of alleles (NA); Effective number of alleles (NA_e_; (Nielsen et al. 2003)); Allelic richness (AR) rarefied to 24 gene copies; Gene diversity corrected for sample size (H_e_; (Nei 1978)); Observed heterozygosity (H_o_); Inbreeding coefficient within populations (R_is_); Genetic differentiation among populations (R_st_; Jost's D); gene flow (N_m_); frequency of null alleles (F_null_; Van Oosterhout et al. 2004); Evidence for an alternative hypothesis for heterozygote excess (HET_exc_) or deficiency (HET_def_; Van Oosterhout et al. 2004).

* Significance levels are denoted, wherever applicable: *p* < 0.05.

** Significance levels are denoted, wherever applicable: *p* < 0.01.

**** Significance levels are denoted, wherever applicable: *p* < 0.0001.

^ns^ Significance levels are denoted, wherever applicable: ns: not significant.

The 402 specimens of *Diphasiastrum digitatum* were genotyped using 13 SSR markers; overall, about 1% of the data was missing after two additional rounds of genotyping, which included 112 data points out of 10,452 (402 samples × 13 loci × 2 alleles per locus; Table S1). Clone correction at the level of either 35 collection sites or 7 subpopulations yielded the same 392 unique MLGs. Attempts at cross‐amplification of the 13 SSRs worked well for *D. digitatum* and showed that the *Diphasiastrum* spp. cross‐amplified much better than the *Lycopodium* spp. (Table S3). Only three SSRs cross‐amplified fully to the related species (Lyco009, Lyco020, Lyco030), and just one more SSR—partially (Lyco016), to only 2 specimens of *Lycopodium* spp. Lyco009 was located in the coding sequence of a promoter‐like binding protein; Lyco016—in a chloroplast/mitochondria S‐adenosylmethionine carrier protein; Lyco020—in an uncharacterized protein with a serine/threonine protein kinase domain; Lyco030—in an uncharacterized putative protein kinase protein.

### 3.2 Hierarchical Population Statistics

Analyses of the *Diphasiastrum digitatum* hierarchical population indices were carried out at the dataset subdivision of 7 subpopulations: ‘VCT_01’ through ‘VCT_04’ encompassing the 32 collection sites of the Virginia Creeper Trail, ‘VT’, ‘NC’, and ‘HR_TN’ (Figure 1). The selected SSRs were powerful at accruing MLGs (Figure S3). Violations of Hardy–Weinberg equilibrium were observed for most of the SSRs in the majority of the subpopulations, likely due to the observed heterozygote deficiency (Tables 1, 2; Figure S4). Based on the analysis of pairwise linkage disequilibrium (LD) between SSRs (Figure S5), the markers used were dispersed across the genome and no linkage‐based bias was detected. The pairwise LD values, represented by $\bar{r}$_*d*_, ranged from −0.02 to 0.18. Notably, all but one pair of SSRs exhibited LD values of < 0.10, indicating generally low level of LD among the markers, which is crucial for accurate genetic mapping and association studies. This independence is particularly important in outcrossing species, where frequent recombination events lead to a breakdown of LD over short genetic distances. Only one pair of SSRs, Lyco022 and Lyco024, showed a demonstrably higher pairwise LD value of 0.18, which in general terms is still considered low. Exploration of the SSR genotyping dataset provided only minor evidence for null alleles presence (Tables 1, 2). All populations and the majority of loci showed null allele frequencies below 0.2, thereby supporting no requirement for genotype adjustments. The null allele frequencies are relatively low and consistent across loci and across populations, which indicated that the data is not biased and therefore reliable (Tables 1, 2). Analyses of heterozygote excess indicated no significant deviations from Hardy–Weinberg Equilibrium (Tables 1, 2). Comparably, low *p*‐values for heterozygote deficiency indicated significant deviations, consistent across loci and populations (Tables 1, 2), suggesting that this overall observation does not compromise our results.

Analyses of the hierarchical population indices showed a substantial excess of the alleles detected over the effective alleles (Table 1). This indicated the predominance of many alleles with low frequency in the actual genotyped dataset. The dataset showed high values of allelic richness and gene diversity (H_e_). The observed heterozygosity (H_o_) was much lower than H_e_, which implies the presence of population structure. Significant levels of inbreeding (F_i_) were also detected, accompanied by low but significant values of $\bar{r}$_*d*_. But, many private alleles (P_a_) were detected, predominantly in the ‘VCT’ subpopulations. Grouping all the collection sites from ‘VCT’ into one subpopulation and re‐analyzing the dataset did not change the population‐wise indices substantially (Table S4). Indeed, it rather highlighted the uniqueness of the ‘VCT’ subpopulation with the majority of private alleles identified it harbored. The combined ‘VCT’ showed comparably higher genetic diversity metrics (NA, NAe, AR) than individual subpopulations, indicative of a broader genetic base when grouped. Similar observations were made in examining individual loci (Table 2), including an excess of alleles detected over the effective alleles, high allelic richness, and H_e_ higher than H_o_. Furthermore, fixation indices proved inconclusive overall for the inbreeding coefficient (*R*_is_ = 0.05) and indicated low differences of allele fixation among subpopulations (*R*_st_ = 0.07). Jost's D = 0.24 indicated high allelic differentiation among subpopulations. Substantial levels of gene flow were detected (*N*_m_ = 2.77).

Analyses of the phylogeographic signals were based on permuting the allele sizes among alleles within each locus. This resulted in retaining the null hypotheses of no phylogeographic patterns within or among the *Diphasiastrum digitatum* subpopulations (permuted *R*_st_ ≈ *F*_st_). As such, the migration rate is not lower than the mutation rate in contributing to the *D. digitatum* diversity, and allele size is non‐informative regarding subpopulation differentiation.

Partitioning of the molecular variance was analyzed both including and excluding the within‐individual levels (Table S5) to assess the contribution of genetic variation within individuals to the overall genetic diversity, thereby providing a more comprehensive view of population structure. This resulted in only minor changes in the molecular variance attributed to between‐subpopulation levels: Within‐samples in 2‐tier AMOVA explained about 91% variance, versus about 57% in 3‐tier AMOVA (Table S5), which indicates that a substantial portion of genetic variation resides within individuals.

### 3.3 Population Structure

The test for isolation‐by‐distance revealed a low value of Mantel's *r* = 0.045 (*p* = 0.072). Adjustment for altitude in the partial‐Mantel test resulted in *r* = 0.036 (*p* = 0.115). This implies that the geographic distance had a negligible effect on the *Diphasiastrum digitatum* differentiation in distant regions, explaining only *r* ^2^ = 0.2% of the genetic variance (Figure S6A,B). The isolation‐by‐environment also analyzed using the Mantel approach yielded *r* = 0.031 (*p* = 0.125), which implied that the environmental differences explained *r* ^2^ = 0.09% of the genetic variance (Figure S6C,D). To further dissect the effects of the environment on *D. digitatum* distribution (Railing and McCarthy 2000; Singleton et al. 2001; Hornbeck et al. 2002; Vogel et al. 2011; Klein 2012), we tried to disentangle which bioclimatic variables had the strongest effect on the genetic clusters identified by structure (see below) within 35 collection sites. The results implied that the variables related directly or indirectly to the water regime had the strongest effects on the assignment to either of the two inferred genetic clusters (Table S6). The variables exhibiting significant associations with the structure‐inferred genetic clusters included Mean Diurnal Temperature Range BIO2, Annual Precipitation BIO12, Precipitation of the Wettest Quarter BIO16, and Precipitation of the Warmest Quarter BIO18. Detailed redundancy analysis (RDA) of these variables and the longitude, latitude, and altitude highlighted the predominant association of altitude with the structure‐inferred genetic clusters, followed by BIO12 and to a comparably lesser extent BIO16 (Figure S7).

**FIGURE 2:**
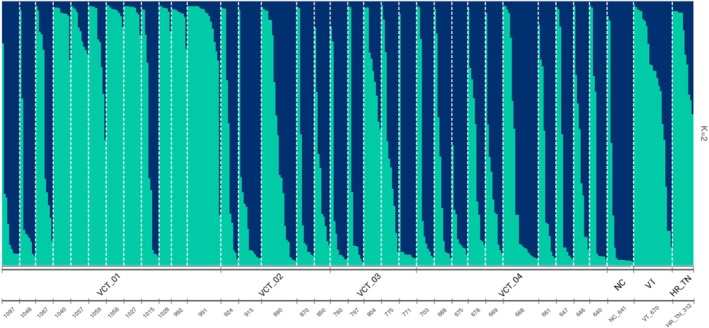
Population structure of *Diphasiastrum digitatum* visualized using structure with the Evanno (Evanno et al. 2005) algorithm identifying *K* = 2 genetic clusters present in the dataset. Individual collection sites are identified at the lower *x*‐axis identified by decreasing altitude, from the starting point of the Virginia Creeper Trail at Whitetop Mountain in VA and grouped by proximity into four populations (‘VCT_01’ through ‘VCT_04’; Table 1). Collection sites from other US states are identified as well (‘NC’: 641 m.a.s.l.; ‘VT’: 670 m.a.s.l.; ‘HR_TN’: 312 m.a.s.l.).

**FIGURE 3:**
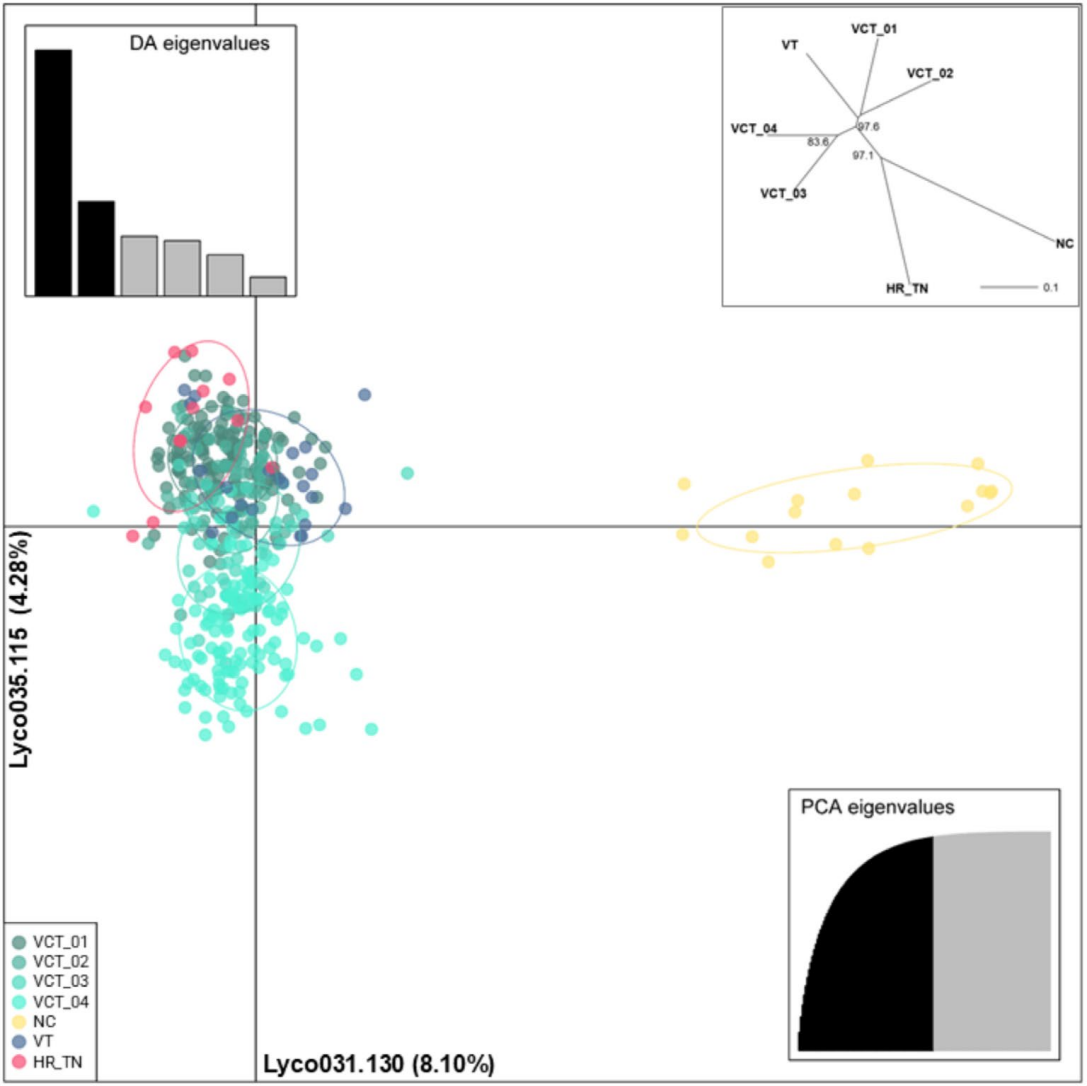
Multivariate analysis of *Diphasiastrum digitatum* genetic diversity. Populations are identified in the legend (bottom left). The number of Principal Components used for the projection is shown in the bottom‐right (*n* = 111), with a darker shade indicating the cumulative variance explained by these Principal Components. The relative eigenvalues for the Discriminant Analysis are shown in the top‐left and indicate the importance of the top two eigenvalues (with darker shade) in effectively separating the groups. Loci and alleles with the highest contributions to explained molecular variance (%) are shown along the *x*‐ and *y*‐axes, respectively. Insert (top‐right): Reticulated tree of pairwise population distances (Prevosti 1974), with bootstrap support of splits indicated (> 70%).

Exploration of the population structure inferred the existence of two genetic clusters (*K* = 2) in the analyzed collection of *Diphasiastrum digitatum* (Figure 2). Strong admixture was present throughout the population, yet the dominant pattern of altitude‐dependent clustering was apparent. This pattern is consistent with the observed high gene flow values and the environmental conditions associated with the prevalent genetic cluster at a given location. The analysis also revealed comparably much smaller peaks at *K* = 6, *K* = 3, and *K* = 9, suggesting additional sub‐structuring. These peaks indicate the presence of more complex genetic patterns that may not be fully captured by the most strongly supported clustering at *K* = 2 (Figure S8). Modest levels of population divergence with an overall *R* ^2^ = 0.25 were detected in the ObStruct analyses (Table S7). Successive iterative removal of the predefined sub‐populations and the inferred clusters indicated a strong impact in only one case: Removal of subpopulation ‘VCT_01’ collected in locations above 991 m a.s.l. of the Whitetop Mountain of the Virginia Creeper Trail, the highest elevation of the trail, substantially decreased the *R* ^2^, thereby indicating that the largest subpopulation also carried the strongest signal for population structure within the dataset. Remarkably, the genetic differences across a fine‐scale elevational gradient were comparable to those observed across a coarse‐scale latitudinal gradient (Figures 3, S8).

All subpopulations except ‘NC’ clustered together using the model‐free multivariate clustering in DAPC (Figure 3). In that group of *Diphasiastrum digitatum* samples, there was a noticeable dispersion among the four ‘VCT’ subpopulations. The ‘VT’ subpopulation was mixed in among the ‘VCT’ subpopulations. Projection of the genetic distances among populations using an unrooted neighbor‐joining tree showed one discrepancy compared with the DAPC results. In the DAPC analyses, the ‘HR_TN’ subpopulation was mixed in with the ‘VCT’ samples and the ‘VT’, whereas the neighbor‐joining analysis placed it as closest to the ‘NC’ (Figure 3; Insert).

Analysis of the genetic barriers among subpopulations identified only two such major barriers. The strongest barrier identified separated the ‘HR_TN’ subpopulation from all the others and was placed by the Barrier program between that and the ‘NC’ subpopulation. The second strongest barrier separated the ‘NC’ subpopulation and the two southern‐most subpopulations from the Virginia Creeper Trail, ‘VCT_01’ and ‘VCT_04’ (Figure 1). The remaining identified barriers were comparably smaller and were therefore dismissed.

### 3.4 Population Demography and Evolutionary History

Analyses of recent evolutionary bottlenecks were carried out on seven subpopulations (‘VCT_01’, ‘VCT_02’, ‘VCT_03’, ‘VCT_04’ from Virginia Creeper Trail and ‘NC’, ‘VT’, ‘HR_TN’) and after grouping the Virginia Creeper Trail data into 1 meta‐population ‘VCT’, as used for the subsequent DIYABC investigations. The cumulative result based on three statistical tests showed no evidence for population bottleneck in either dataset subdivision. This is despite the fact that individual tests had occasional departures from the expected mutation‐drift equilibrium, that is, showed population bottlenecks (Table S8).

**FIGURE 4:**
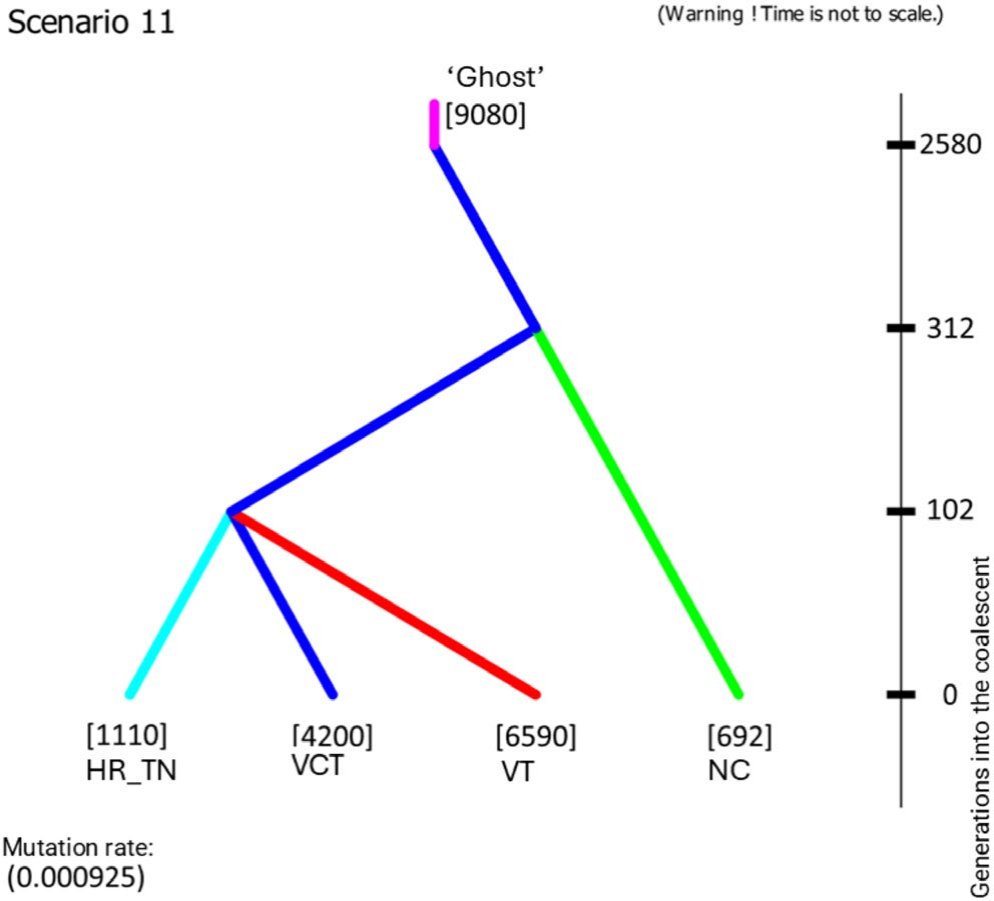
The best‐supported evolutionary scenario as per the DIYABC analyses. Population names are identified at each leaf and correspond to Virginia Creeper Trail (‘VCT’); North Carolina (‘NC’); Vermont (‘VT’); and Highland Rim, Tennessee (‘HR_TN’). Estimated medians of effective population sizes are given in brackets for each population. The median of the estimated mutation rate is presented in the bottom‐left corner. Estimated medians of split times (in generations) into the coalescent are presented along the right‐hand time schematic.

DIYABC analyses indicated strong support for scenario 11. This scenario assumed an unsampled starting population “Ghost” from which the ‘VCT’ population diverged about 2580 generations into the coalescent. At about 312 generations into the coalescent, that population gave rise to the ‘NC’ population, followed by a concurrent split at about 102 generations into the coalescent to the ‘VT’ and the ‘HR_TN’ populations (Figure 4). The overall mutation rate was calculated at about 10^−3^ mutations per generation per locus, with effective population sizes ranging from about 692 for the ‘NC’ population to about 9080 for the unsampled ‘Ghost’ starting population (Figure 4; Table S9). The second strongest‐supported scenario, Scenario 10, was similar to Scenario 11, except that it had the ‘NC’ and ‘HR_TN’ populations switch places (Figure S9). The accepted scenario 11 and the calculated times of splits suggested the species expansion from the southern refugium starting after the Last Glacial Maximum. Analyses of bias in the calculated parameters versus the observed values on prior/posterior parameter spaces showed acceptable values. The genetic information provided by the data resulted in smaller accuracy values of Relative Root Mean Integrated Square Error (RRMISE) and Relative Mean Absolute Deviation (RMeanAD) compared to analyses that did not incorporate this information (Table S9). Furthermore, the global prior predictive error between Scenario 11 and 10 was 0.141 (logistic: 0.123); the posterior global predictive error was 0.141 (logistic: 0.123). The calculated prior based error analysis where PODs are drawn from parameter prior distributions under Scenario 10 (or 11) reached the following values: Type I error of 0.158 (logistic: 0.137) and Type II error of 0.133 (logistic: 0.127). Analyses of bias, precision, and confidence thus support the selection of evolutionary Scenario 11.

### 3.5 Ecological Niche Modeling Using MaxEnt

**FIGURE 5:**
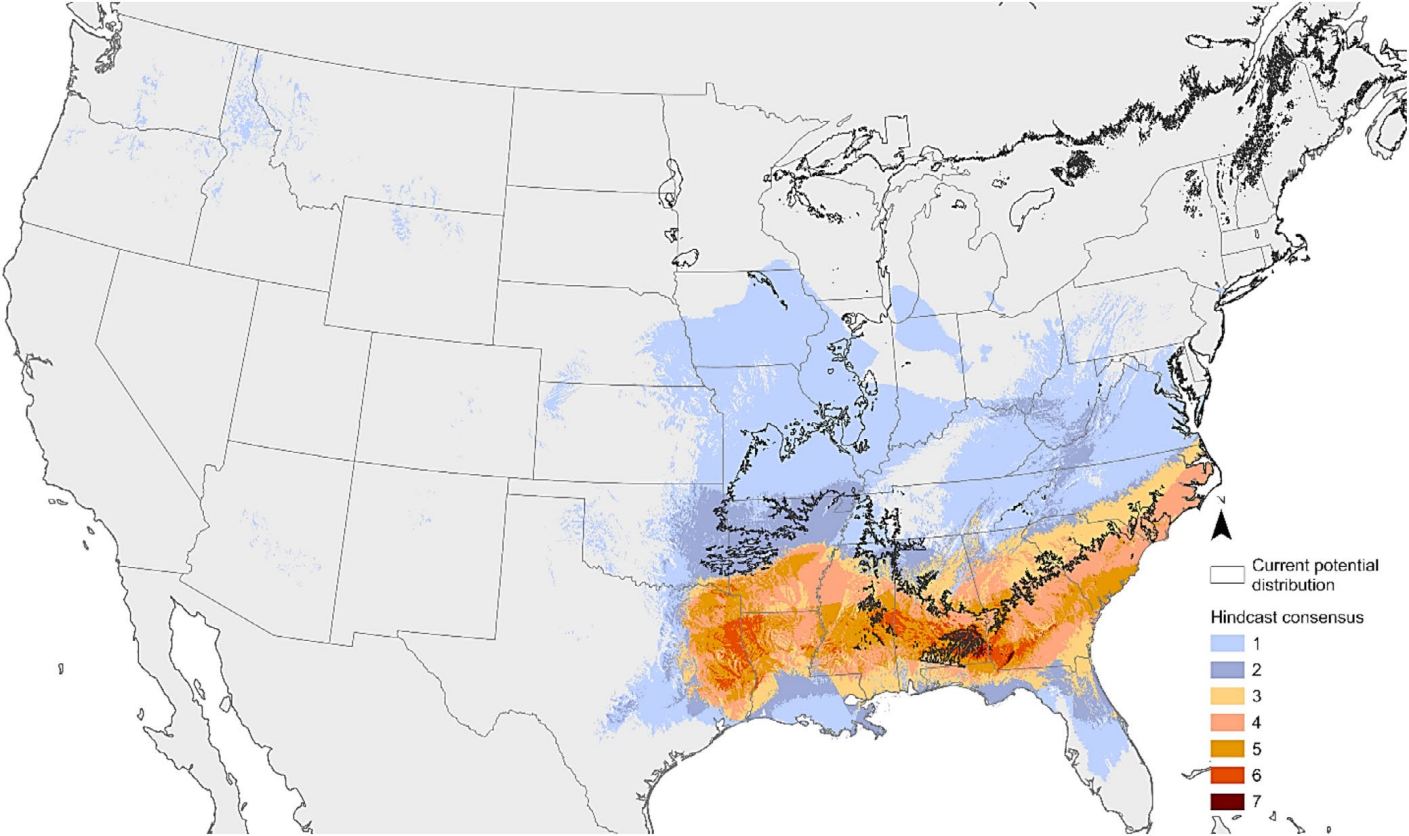
Ecological niche modeling for *Diphasiastrum digitatum* using MaxEnt. Current plant occurrences and current potential distribution (black line) were used as input for the modeling of spread under seven historical scenarios, reaching back to the Last Glacial Maximum. Historical occurrences present under multiple overlapping scenarios are shown coded by color, from 1 scenario (light blue) to all 7 (dark red).

After removing duplicate occurrence records of *Diphasiastrum digitatum* in GBIF falling within the same 1 × 1 km pixel, the presence dataset was reduced from 1065 records (dated 1976 to 2015) to 837 records (Figure S10). The MaxEnt model evaluated the individual contributions of the 19 bioclim variable predictors to the model accuracy gain. Nine variables each contributed at least 3% and cumulatively contributed 86% to the model accuracy gain. After the removal of correlated, potentially confounding variables, the remaining 6 bioclim variables (Diurnal Temperature Range BIO2; Temperature Seasonality BIO4; Mean Temperature of Wettest Quarter BIO8; Mean Temperature of Warmest Quarter BIO10; Precipitation Seasonality BIO15; and Precipitation of the Warmest Quarter BIO18) had correlation coefficients < 0.7. This subset of 6 variables was used to calibrate new MaxEnt models that were then projected onto 7 LGM climate reconstructions, each with the same subset of 6 variables. Most of the LGM climate datasets showed minimal climatic suitability for the species during LGM. Only one LGM climate reconstruction showed considerable climatic suitability: MRI_MRI‐CGCM3 (data not shown). BIO2 and BIO4 were limiting the model projections for several LGM reconstructions because these variables can substantially influence the climatic suitability for species modeling. During the LGM, climatic conditions were markedly different from present‐day conditions, with more extreme temperature variations and seasonality. As such, MaxEnt was then re‐run for current and LGM distributions without these bioclim predictors. Under such projections, the climatic suitability for the species during LGM varied greatly depending on the LGM climate reconstruction (Figure 5).

The hindcast reconstructions point to the expansion of the species after the LGM, based on the consensus from 7 reconstruction models (Figure 5). Furthermore, in agreement with the evolutionary scenario based on the SSR data, these results suggest that *Diphasiastrum digitatum* spread after the LGM from its southern refugium into the current distribution across a major part of the eastern US.

## 4 Discussion

Our analyses of the molecular diversity in *Diphasiastrum digitatum* in the context of the species evolutionary history and ecological niche supported our working hypothesis of the ecological factors shaping the species diversity. Development of the transcriptome‐based SSR markers, their experimental validation, and application to the genotyping of the collections of *D. digitatum* provided ample data for downstream analysis that enriched our knowledge of this understudied lycopod species (Petlewski et al. 2022).

We developed a new molecular toolbox based on a de novo approach because our initial attempts at cross‐amplification using 11 genomic DNA SSRs developed for a related species, *Lycopodium fordii* Bak. (Huang et al. 2008) were not successful (Nowicki et al., unpublished manuscript). This may signify the substantial taxonomic distance between the Asia‐native species and *Diphasiastrum digitatum* and thus supports the currently recognized taxonomic placement of the latter (Aagaard et al. 2009; Su et al. 2021). The more distantly related *D. sabinifolium* (Aagaard et al. 2009; Su et al. 2021) amplified with only one of our SSRs. The underlying reason for the observed cross‐amplification to several related species native to North America is likely due to the microsatellite development approach. One of our objectives—to analyze *D. digitatum* diversity using our markers and to test their effectiveness for genotyping of related lycopods or for other taxonomic and phylogenetic purposes (Petlewski et al. 2022)—still awaits complete validation. However, our current results indicate they would be useful for related lycopods. Only a handful of studies have developed and deployed SSRs for population‐scale analyses of members of Lycopodiales (Huang et al. 2008; Aagaard et al. 2009; Luo et al. 2010; Schnittler et al. 2019). Previously, several groups attempted diversity analyses based on isoenzyme patterns (Levin and Crepet 1973; Soltis and Soltis 1988). Other molecular approaches included amplified fragment length polymorphism (Huang and He 2010; Schnittler et al. 2019) or more recently, next‐generation sequencing and phylogenomic analyses (Su et al. 2021; Petlewski et al. 2022). Based on our current knowledge, our study represents one of the largest collections of molecularly analyzed Lycopodiales.

In the analyses of diversity and evolutionary history of *Diphasiastrum digitatum* , we exhaustively sampled the population of the Virginia Creeper National Recreation Trail in Virginia, USA, with other locations in the USA sampled less intensively for added phylogeographic context. Our sampling scheme of at least 5 m apart was set up to avoid genotyping the same individuals, as the growth rates in a related plant, *Spinulum annotinum* (L.) A. Haines, were estimated at about 1 m across 20 years (Callaghan 1980; Callaghan et al. 1986). This fact was further underscored in the retention of almost 98% of the collected samples as unique MLGs, even after binning the original genotypic data into allelic classes, which inherently tends to decrease the number of unique MLGs.

Our SSRs effectively captured MLGs, which indicated their suitability for assessing genetic diversity. The presence of inbreeding, private alleles in ‘VCT’, and the low‐frequency alleles collectively suggested population structure, local adaptation, and unique evolutionary histories. The observed heterozygosity, being lower than the expected heterozygosity, indicated the influence of population structure, possibly due to inbreeding. Our study supported the rejection (Soltis and Soltis 1988, 1989; Schnittler et al. 2019) of the long‐standing notion of gametophytic selfing in Lycopodiales (Levin and Crepet 1973; Wittig et al. 2007) with results suggesting substantial levels of heterozygosity in our genotyping dataset both at population‐ and locus‐wise levels, despite some inbreeding. Our data add to the evidence that outcrossing utilized as a reproductive option may be operative in Lycopods, and more specifically—in *D. digitatum* (Soltis and Soltis 1988; Haufler et al. 2016; Schnittler et al. 2019), with only one of the analyzed SSRs (Lyco009) yielding no heterozygotes and our markers distributed throughout the genome. Specifically, *D. digitatum* shows a clear outcrossing reproductive pattern. The substantial levels of heterozygosity observed suggest that *D. digitatum* relies on cross‐fertilization rather than self‐fertilization. Such outcrossing reproductive strategy enhances the adaptability and resilience of populations by increasing genetic variation, which is crucial for responding to environmental changes and pressures (Yin and Meicenheimer 2017; Schnittler et al. 2019). High gene flow and genetic diversity within *D. digitatum* populations further support the outcrossing hypothesis, aligning with the broader understanding of reproductive strategies in Lycopodiales (Railing and McCarthy 2000; Yin and Meicenheimer 2017), thereby providing new insights into their evolutionary and ecological dynamics. The identity of the single purely homozygous locus Lyco009 is unclear (no BLAST results were accrued for Lycopodiales); with the ongoing development of plastid and mitochondrial genomic resources for this species, the possible location of Lyco009 in those genomes would yield haploid—and thus 100% homozygous—PCR products.

Our findings on the genetic diversity and population structure of *Diphasiastrum digitatum* can be compared with several recent studies on other *Diphasiastrum* species. Investigations of three European *Diphasiastrum* species and their hybrids using plastidial microsatellites, nuclear markers, and AFLP found that the hybrid taxa independently formed F_1_ crosses without evidence of recent backcrossing; this suggested limited fertility of these hybrids (Schnittler et al. 2019). In contrast, our study revealed ongoing gene flow and recent divergence, with evidence of substantial heterozygosity and outcrossing. Examinations of *D. alpinum* and *D. sitchense* from the Russian Far East, along with their hybrid *D. × takedae*, used chloroplast microsatellites, nuclear markers, and genotyping by sequencing, and detected introgression and backcrossing in the hybrid taxon indicative of some fertility (Bog et al. 2022). This differs from our findings of high levels of gene flow but no direct evidence of hybridization or introgression with closely related species. Studies of the genetic diversity and structure of *D. alpinum* across its Eurasian range using plastidial and mitochondrial microsatellites and nuclear markers found distinct genetic clusters corresponding to geographical regions, with evidence of postglacial recolonization from multiple refugia (Schnittler et al. 2024). This pattern is similar to our observations with detected genetic structuring influenced by environmental factors and evidence of expansion from southern refugia after the Last Glacial Maximum. These comparisons highlight the diverse evolutionary patterns extant within the genus *Diphasiastrum*, which range from strict reproductive isolation in hybrid taxa to ongoing gene flow and potential introgression. Our study on *D. digitatum* adds to this growing body of knowledge and emphasizes the importance of species‐specific investigations to understand the complex evolutionary dynamics in this group of lycophytes.

The complex interplay of migration and mutation in *Diphasiastrum digitatum* revealed ongoing gene flow and recent divergence through several key observations. High levels of genetic diversity within populations indicated ongoing gene flow, which suggested that individuals from different populations are interbreeding. The wind‐mediated dispersal is likely a significant factor contributing to the high migration rates observed. Additionally, habitat connectivity, such as large continuous tracts of coniferous forests, can enhance spore dispersal and establishment, further promoting gene flow. Human activities, including logging and land clearing, may also inadvertently aid in spore dispersal by creating new habitats or moving soil and plant material. The phylogenetic trees constructed from genetic data show closely related lineages, which is consistent with recent divergence, thereby indicating that the populations have not been separated long enough to accumulate substantial genetic differences. The distribution of genetic markers across different geographic locations showed patterns of migration and gene flow. This is evident from the lack of strong genetic differentiation between populations, indicating that there is movement and interbreeding between them. These factors together suggest that *D. digitatum* populations are not isolated but are instead experiencing ongoing gene flow, which contributes to their genetic diversity and recent divergence. Similarly, Hardy–Weinberg deviations, inbreeding, and gene flow hinted at adaptive processes and ongoing divergence (Schnittler et al. 2019). AMOVA provided evidence of within‐subpopulation gene flow, possibly influencing genetic homogenization and diversity maintenance (Soltis and Soltis 1988; Huang and He 2010; Schnittler et al. 2019). Although genetic homogenization and maintaining diversity might seem counterintuitive, they can coexist in this context. Genetic homogenization acts to reduce the genetic differences between subpopulations due to gene flow, whereas maintaining diversity signifies the overall genetic variability within the entire population. In the case of *D. digitatum* , gene flow within subpopulations can lead to homogenization at the subpopulation level, but the overall genetic diversity of the species is maintained through the introduction of new alleles and the mixing of genetic material across the broader population (Schnittler et al. 2019; Landis et al. 2024). The patterns of allelic richness, genetic diversity, and gene flow reflected the dynamic genetic processes shaped by migration, selection, and genetic exchange. The high values for allelic richness and gene diversity suggest a rich genetic reservoir within populations, whereas substantial levels of inbreeding and private alleles indicate potential evolutionary processes that shape population dynamics.

The detection of population‐specific alleles may indicate the importance of local adaptation and genetic uniqueness. Under such conditions, certain alleles confer a selective advantage in specific environmental conditions. For instance, environmental variables related to water regime, such as annual precipitation and precipitation of the warmest quarter, were significantly associated with genetic clustering. These environmental factors could directly influence the fitness of different genotypes, leading to local adaptation (Ricklefs 2006; Shao et al. 2019). In small populations, genetic drift can lead to random changes in allele frequencies, contributing to genetic differentiation. This effect might be more pronounced in isolated populations or those that have experienced recent reductions in size (Levin and Crepet 1973; Soltis and Soltis 1988). Although genetic drift is a stochastic process, its impact can be significant, especially in populations with limited gene flow.

The analyses of population structure helped elucidate the patterns of genetic differentiation and gene flow. The identification of genetic clusters, associated with environmental correlations, provided insights into the possible drivers of population divergence and adaptation. The observed admixture and geographic patterns of clustering suggested a dynamic interplay between historical processes and contemporary environmental factors that shape the population structure of *Diphasiastrum digitatum* . The DIYABC analyses further supported these findings by suggesting that the species has experienced dynamic changes over time, influenced by both historical and contemporary factors. As a late successional species due to its prolonged gametophytic development (Eames 1942; Hornbeck et al. 2002; Aagaard et al. 2009), the presence of *D. digitatum* can serve as an important indicator for the lack of recent major disturbance (Singleton et al. 2001; Vogel et al. 2011), especially in relation to the water‐related climatic factors, here uncovered as crucial for the species. This species thrives in environments where the water regime is stable and consistent, which is crucial for its prolonged gametophytic development (Wagner 1990; Gilman 2001; Whittier 2003; Rimgailė‐Voicik 2017). Fluctuations in water availability can negatively impact the delicate balance required for its development, thereby leading to reduced population viability. Therefore, the presence of *D. digitatum* makes it a valuable bioindicator for assessing the health of forest ecosystems, particularly in relation to water availability and soil moisture.

Our findings on the genetic diversity and population structure of *Diphasiastrum digitatum* revealed a strong differentiation of the ‘NC’ population from the others. This unexpected result warrants further discussion. Although our study did not deposit herbarium vouchers, which could have aided in morphological verification, we can speculate on this observation using our molecular data and previous studies. The strong genetic differentiation of the ‘NC’ population could be attributed to several factors. It may represent a genetically distinct lineage within *D. digitatum* , possibly resulting from long‐term isolation or adaptation to specific environmental conditions in the region. Our genotyping data reveal one unique allele in the ‘NC’ population which lends some support to this notion. Alternatively, it could potentially represent a morphologically cryptic taxon, such as a hybrid between *D. digitatum* and *D. tristachyum* , which is known to be common in the mid‐Atlantic region and can be difficult to distinguish morphologically from *D. digitatum* (Hanušová et al. 2014). Examination of our cross‐amplification results and comparisons with the findings on European *Diphasiastrum* taxa (Schnittler et al. 2019) show only slight differences in SSR amplification patterns between *D. digitatum* and *D. tristachyum* , with all loci amplifying successfully in both species. This high degree of cross‐amplification is consistent with the close relationship between these taxa but does not conclusively rule out the possibility of hybrid sampling. The hybrids in European *Diphasiastrum* consistently displayed one allele from each parent for the nuclear markers (Schnittler et al. 2019), whereas our data for ‘NC’ show a mix of alleles shared with other populations and one unique private allele. This pattern does not strongly indicate a hybrid origin but suggests a complex evolutionary history. To definitively resolve the taxonomic status of the ‘NC’ population, future studies should incorporate herbarium vouchers, detailed morphological analyses, and additional molecular markers specifically designed to distinguish between *D. digitatum* and *D. tristachyum* , including plastidial or mitochondrial ones (Bog et al. 2022). Flow cytometry, which has been successfully used to identify *Diphasiastrum* hybrids (Bennert et al. 2011), could also provide valuable insights. Furthermore, expanding the sampling to include more populations from the mid‐Atlantic region might help determine whether this genetic differentiation is localized or part of a broader pattern. Notably, our ‘NC’ sample size was smaller than that of some other populations, which calls for cautious interpretation and further sampling to confirm these patterns.

Analyses of evolutionary bottlenecks showed minor evidence of variable population dynamics, suggesting possible environmental influences. The accepted DIYABC scenario aligned with our hypothesis of a southern refugium that played a key role in the species' evolutionary history. That refugium was previously confirmed in a southeastern forest tree species (Ony et al. 2021). In agreement with that notion, the calculated expansion timing reflected resilience and adaptive responses to changing environments in *Diphasiastrum digitatum* habitat. Our DIYABC analyses implied that *D. digitatum* populations expanded from a southern refugium after the LGM into new areas, which demonstrates the species' ability to adapt to changing environmental conditions. Maintenance of a robust genetic pool is crucial for adaptability, as it provides the raw material for natural selection to act upon, enabling the species to respond to environmental changes (Schnittler et al. 2019; Landis et al. 2024). Lack of population bottlenecks indicates that *D. digitatum* populations have been relatively stable, which, combined with the observed admixture and geographic clustering, suggests that the species has been able to maintain its population structure and adapt to varying environmental pressures. The DIYABC analyses can be sensitive to the unbalanced sampling of populations, particularly the possible overrepresentation of the ‘VCT’. To mitigate this potential bias, we conducted sensitivity analyses and model checking to critically assess our results. This ensured that our evolutionary and demographic inferences are robust and reliable, despite the initial unbalanced sampling. Alternative approaches could utilize more robust sampling (occasionally biologically impossible), subsampling to approximately comparable population sizes (potentially missing parts of valuable genetic information), or data weighting (which can introduce its own biases, potentially skewing the results in unintended ways).

Following the retreat of the ice sheets at the end of the LGM, *Diphasiastrum digitatum* populations began to expand northward from the southern refugia. This post‐glacial expansion is supported by genetic evidence indicating high levels of gene flow and low genetic differentiation among populations across eastern North America. The genetic clustering patterns observed in our study, along with ecological niche modeling, suggest that *D. digitatum* spread from its southern refugium into its current distribution range. The timeline of this expansion can be inferred from genetic data and ecological modeling. Our DIYABC analyses indicate that the divergence of *D. digitatum* populations occurred approximately 2580 generations ago, with subsequent splits leading to the formation of distinct regional populations. This timeline aligns with the post‐LGM period, supporting the hypothesis of a southern refugium and subsequent northward expansion.

Ecological modeling further underscored the environmental correlation with *D. digitatum* distribution and genetic diversity. Isolation‐by‐environment yielded results comparable to isolation‐by‐distance, but the additional insights highlighted the importance of ecological factors for the species biology, as indicated in other relevant studies (Gilman 2001; Singleton et al. 2001; Ricklefs 2006). More specifically, water‐related WorldClim variables emerged as the key factors associated with genetic clustering patterns, based on the x2y analyses. Genetic barriers and ecological niche modeling alignment also suggested environmental influence on the species' genetic structure. Altitude‐dependent genetic clustering visualized by structure further supported the ecological niche modeling, thereby emphasizing altitude and water availability as the shapers of the observed genetic patterns. These findings have implications for conservation, evolutionary understanding, and management of *D. digitatum* (Gilman 2001; Hornbeck et al. 2002). For example, the detected private alleles highlighted the need for the conservation of unique lineages present in the Virginia Creeper Trail. The associations between genetic clustering and environmental factors necessitate habitat preservation and restoration (Hornbeck et al. 2002). Evidence of expansion from southern refugium after the LGM echoes climate resilience of *D. digitatum* , similar to circumboreal distribution of related lycopods (Klein 2012; Petlewski et al. 2022). These findings contribute to understanding the species' evolutionary trajectory and adaptive potential in response to past climate fluctuations. Ecological niche modeling provided insights into the species' past distribution dynamics and potential responses to climate change. By identifying historical refugia and patterns of expansion, we can infer the species' resilience and adaptability to past climate fluctuations. Such historical perspectives can suggest how the species might cope with similar changes in the future (Chevalier et al. 2024). The genetic diversity and stability observed in *D. digitatum* populations indicate a robust capacity to adapt to changing conditions. That potential for adaptability is crucial for predicting how the species might respond to future climate scenarios (Li et al. 2023). Understanding the environmental preferences and tolerances of *D. digitatum* helps in predicting its potential range shifts under different climate scenarios. For example, if a species thrives in moist, shaded environments, changes in precipitation and temperature patterns could notably impact its distribution (Zurell et al. 2024). Similar studies on other plant species have shown that past distribution dynamics can provide valuable insights into future responses to climate change. For instance, research on forest tree species in southeastern forests has highlighted the importance of historical refugia in shaping current distribution patterns and potential future shifts (Ony et al. 2021; Franklin 2024). By integrating current and past climate data, our models offer predictions of historical habitat suitability and highlight regions of refuge and expansion during climatic fluctuations. These findings contribute to broader discussions on species' resilience and vulnerability to environmental change (Hornbeck et al. 2002; Klein 2012; Petlewski et al. 2022; Treder et al. 2023).

Although our results indicate a substantial correlation between genetic population structure and environmental variables in shaping the extant diversity of *Diphasisatrum digitatum*, it is crucial to recognize that such patterns could arise from other unrelated mechanisms. It is plausible that environmental selection directly favors certain genotypes in specific habitats. For example, water‐related variables such as annual precipitation and precipitation of the warmest quarter were significantly associated with genetic clustering. These environmental factors could directly influence the fitness of different genotypes, leading to local adaptation (Ricklefs 2006; Shao et al. 2019). Alternatively, the observed genetic structure could result from indirect effects of environmental variation. Environmental factors might shape the expression of abiotic or biotic factors that act as the actual selective forces. For instance, variations in water availability could influence soil properties, microbial communities, or the presence of symbiotic fungi, which in turn affects the growth and survival of *D. digitatum* (Horn et al. 2013; Winther and Friedman 2008). High levels of gene flow and migration can also shape genetic structure. The wind‐mediated dispersal of spores facilitates gene flow between distant populations, thereby reducing genetic differentiation. However, barriers to gene flow, such as geographic features or habitat fragmentation, could lead to the formation of distinct genetic clusters (Schnittler et al. 2019; Landis et al. 2024). Understanding the potential causes of genetic population structure is essential for informing conservation strategies. If environmental selection is a primary driver, conservation efforts should focus on preserving the specific habitats and environmental conditions that support local adaptations. Conversely, if historical demographic events or gene flow are more influential, maintaining connectivity between populations and protecting genetic diversity across the species' range would be crucial (Cadotte et al. 2008; Gilman 2001). Further research is needed to disentangle the relative contributions of these various mechanisms and scenarios. Experimental studies that manipulate environmental conditions and track genetic changes over time could provide insights into the role of selection. Additionally, genomic approaches that identify specific genes or genomic regions under selection could help clarify the genetic basis of local adaptation (Su et al. 2021; Petlewski et al. 2022).

Collectively, our multifaceted analysis informs conservation, understanding evolution, and species management in *Diphasiastrum digitatum* . These results contribute to our understanding of the evolutionary dynamics of the “phylogenetic relic[s]” (Levin and Crepet 1973; Su et al. 2021) and provide a foundation for further research on the conservation and management of lycopods. By identifying populations with high genetic diversity, we highlight key areas for habitat protection and restoration efforts. Understanding the genetic structure and connectivity between populations allows for the development of strategies to enhance habitat corridors and reduce inbreeding risks. Integrated approaches are pivotal in safeguarding species in a changing world. Further investigations are warranted to explore the functional significance of the identified SSR loci and to elucidate the mechanisms underlying the observed population dynamics and adaptation in *D. digitatum* .

## 5 Conclusions

Our comprehensive study delved into the molecular diversity of *Diphasiastrum digitatum* across various locations from its native range in the Eastern US, thereby providing crucial insights into the evolutionary history and ecological niche of the relatively understudied clubmoss plants—a member of the lycophyte lineage of plants (Levin and Crepet 1973; Su et al. 2021). Development and validation of transcriptome‐based SSRs filled a critical knowledge gap but also highlighted the taxonomic distance between *D. digitatum* and related lycopods. Isolation‐by‐distance analysis indicated that geographic distance had a minimal effect on genetic differentiation, whereas environmental variables related to water regime were associated with genetic variance. Ecological niche modeling showed a post‐Last Glacial Maximum expansion of *D. digitatum* from southern refugia, corroborating a similar evolutionary scenario based on our SSR microsatellite data. Our findings underscored the complexity of genetic processes that shape the species' dynamics, including population structure, migration, and adaptation. Ecological niche modeling analyses revealed the possible influence of environmental factors on genetic patterns, with implications for conservation and management strategies, such as highlighting populations for preferential monitoring and conservation. Overall, our study enhanced the understanding of lycopods' evolutionary dynamics and provided a platform for further research in species conservation and management, emphasizing the importance of integrated approaches in safeguarding biodiversity in a changing world.

## Author Contributions

**Marcin Nowicki:** funding acquisition (equal), methodology (lead), visualization (lead), writing – original draft (lead), writing – review and editing (equal). **Logan C. Houston:** data curation (equal), investigation (lead), methodology (supporting), resources (equal), validation (equal). **Sarah L. Boggess:** data curation (equal), formal analysis (lead), investigation (supporting), project administration (lead), writing – original draft (equal), writing – review and editing (equal). **Matthew L. Huff:** formal analysis (supporting), investigation (equal), methodology (equal), writing – original draft (equal), writing – review and editing (equal). **Margaret E. Staton:** conceptualization (supporting), funding acquisition (supporting), project administration (equal), resources (equal), supervision (equal). **Robert N. Trigiano:** conceptualization (lead), funding acquisition (lead), resources (equal), supervision (equal), writing – original draft (equal), writing – review and editing (equal).

## Conflicts of Interest

The authors declare no conflicts of interest.

## Supporting information


Figure S1.

Table S1.

## Data Availability

The genotyping and geospatial data underlying the presented analyses is presented in full in Supplementary Tables. The RNA data used for the generation of *Diphasiastrum digitatum* transcriptome is publicly available (https://www.ncbi.nlm.nih.gov/sra/?term=ERR2040872).
