# Supplementary material for: Integrative Analysis of Diphasiastrum digitatum Holub: Unveiling Genetic Variation and Ecological Adaptations for Sustainable Ecosystem Management
Source: Ecol Evol. 2025 Mar 13;15(3):e71079. doi: 10.1002/ece3.71079 (PMC11906285; doi:10.1002/ece3.71079)

Supplementary Figure F1: *Diphasiastrum digitatum* in natural habitat. Subpopulation ‘VCT_01’, specifically at the sampling site of 1059 m.a.s.l. (GPS: 36.60826, -81.63655) is shown. Picture date and collection date was 2018-06-02.


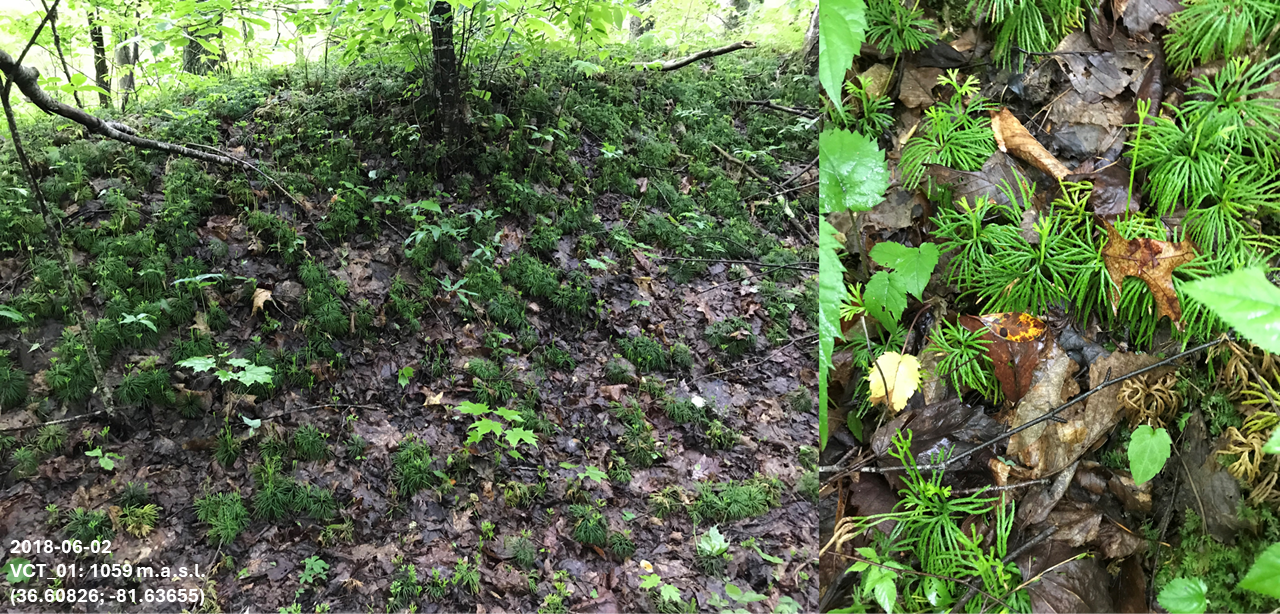


Supplementary Figure F2: Abundance of microsatellite markers (SSRs) detected in the *Diphasiastrum digitatum* transcriptome. (**A**) The main graph shows the overall number of SSRs detected (**navy blue**) and the number of SSRs with primers detected (**mint green**). The X axis is organized by the decreasing counts of the repeated motifs among the SSRs. (**B**) In similar colors, SSRs are grouped by the motif size – from 2 bp to 4 bp.


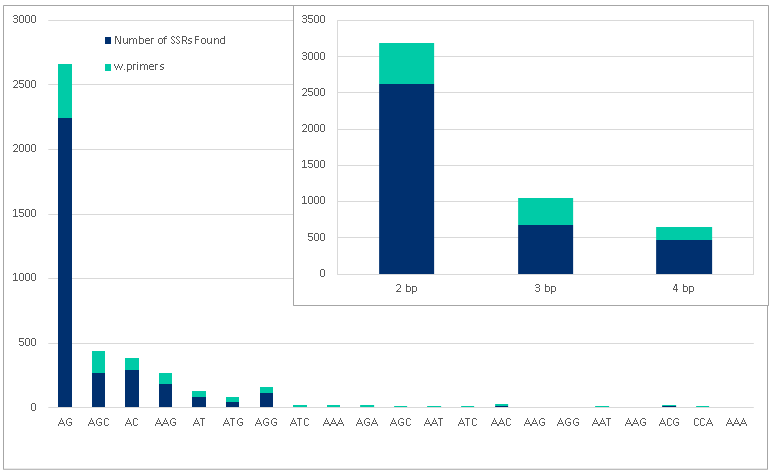


**B**

**A**

Supplementary Figure F3: Genotype accumulation curve indicates the robustness of the developed *Diphasiastrum digitatum* selected SSRs in detecting the diversity among the analyzed multi-locus genotypes. About 12 markers are needed to approximate the saturation of such detection.


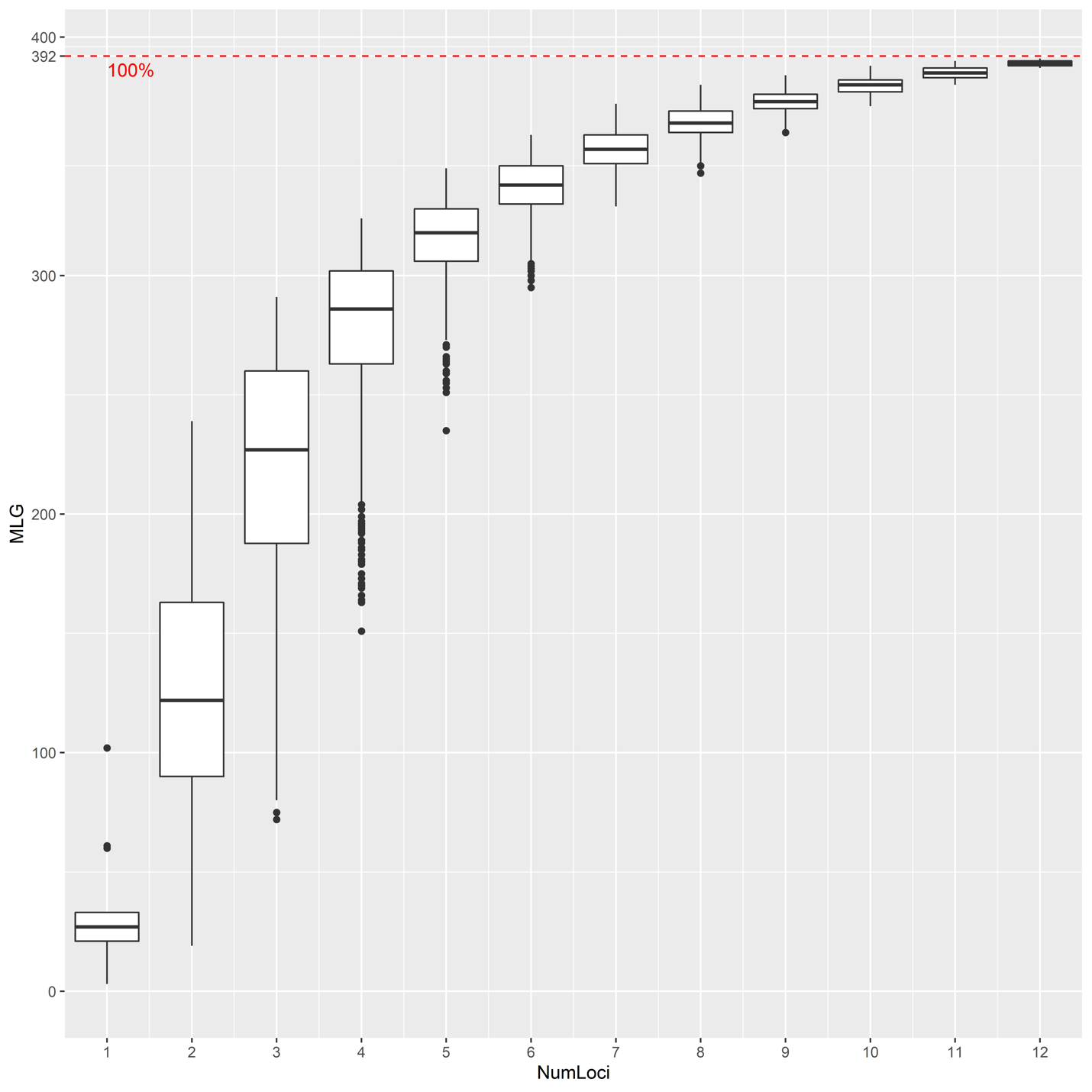


Supplementary Figure F4: Violations of the 13 *Diphasiastrum digitatum* microsatellites (SSRs) of the Hardy-Weinberg equilibrium. SSR loci are presented along the Y-axis, whereas the populations analyzed – along the X-axis. Color of the intercepts (legend on the right) denotes the violations of the expected equilibrium (pink).
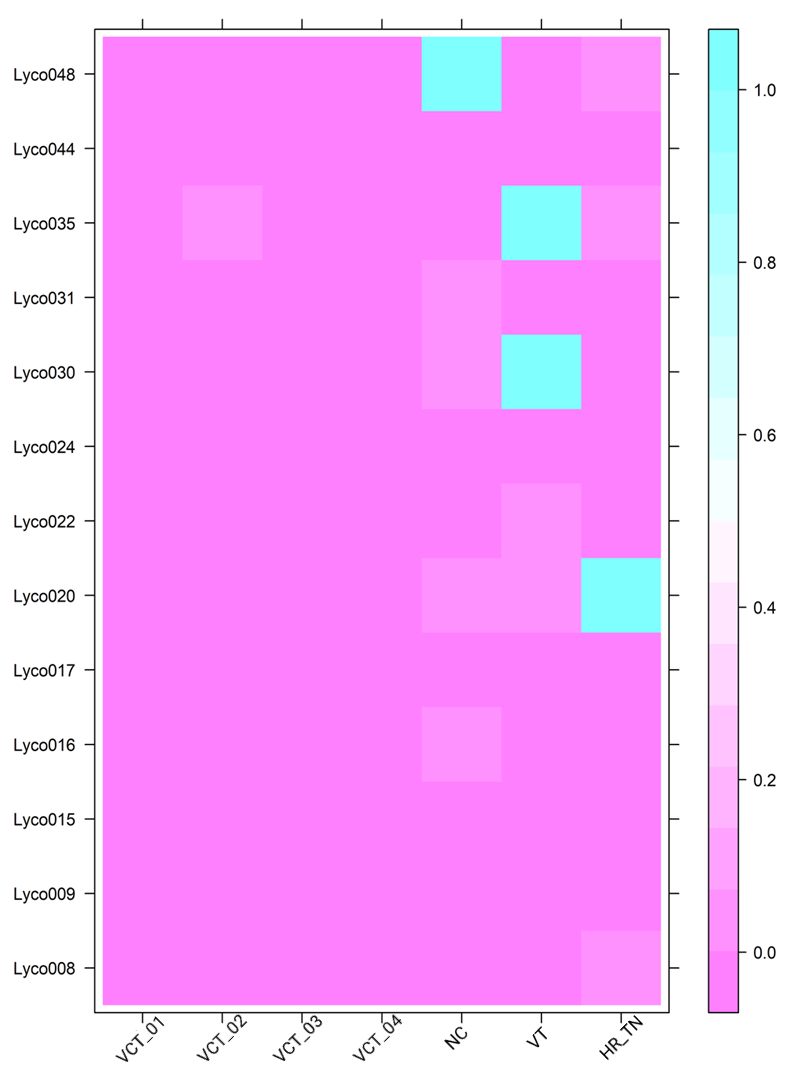


Supplementary Figure F5: Pairwise linkage disequilibrium among the 13 *Diphasiastrum digitatum* microsatellite markers. Shades of gray at each intercept show the strength of such linkage, as explained in the right-hand legend.


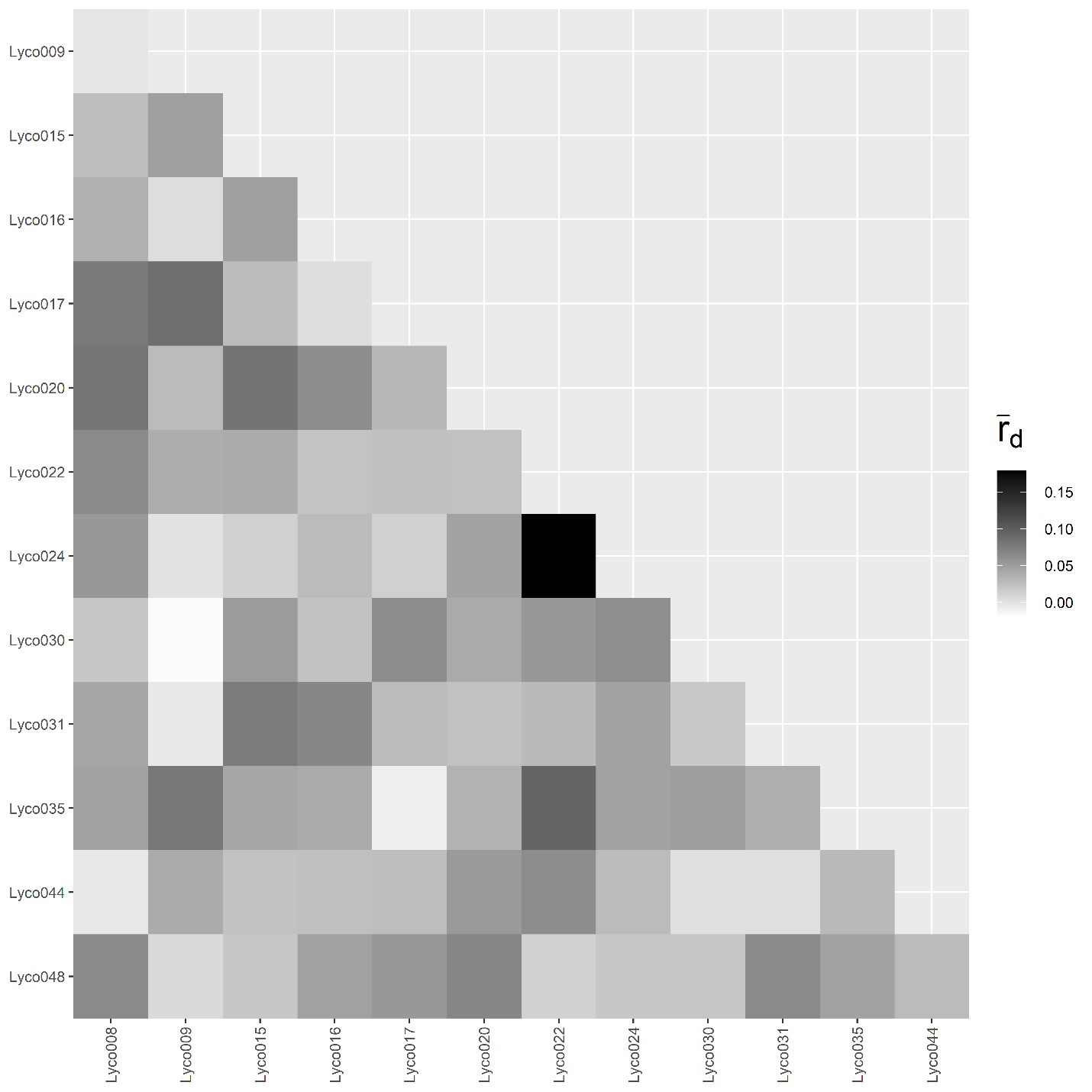


Supplementary Figure F6: Mantel tests for Isolation-by-Distance (A;B) and Isolation-by-Environment (C,D) in the collection of *Diphasiastrum digitatum*. Mantel scores for correlation of genetic distance (Y-axis) and geographic distance (A) or ecological distance (C) are shown in bottom-right corners. For (A), the r’ was generated by standardizing the data by altitude (Suppl.Table T1). Correlograms were developed in similar manner, to give more insights into how Mantel’s r scores are variable across geographical (B) or ecological (D) distances.
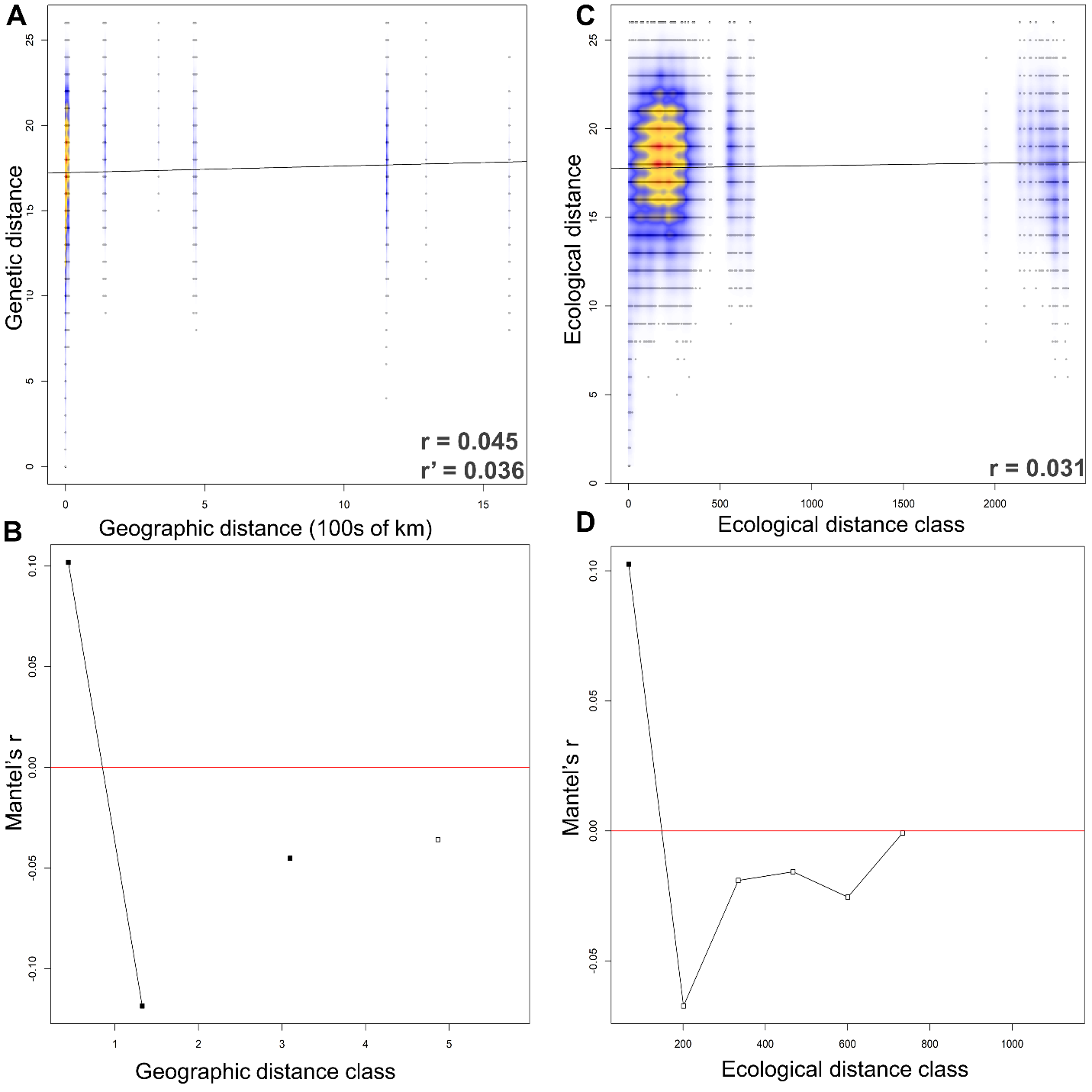


Supplementary Figure F7: Redundancy Analysis (RDA) Biplots showing the distribution of populations and the contributions of WorldClim environmental variables. Populations are color-coded based on their group, with ‘VCT’ collections sites sharing similar major colors but differing in shades by suffixes (01 through 04). ‘NC’, ‘VT’, and ‘HRTN’ populations are represented by distinct colors from the Viridis palette for visual accessibility. For each collection site, the respective genetic clusters inferred by Structure are represented by different shapes: filled squares for Cluster 1 and stars for Cluster 2. Insert: Zoomed-in Redundancy Analysis Biplot focusing on the -2.5 to 2.5 range on PC1 and PC2 to better visualize the contributions of WorldClim environmental variables and populations within that range.


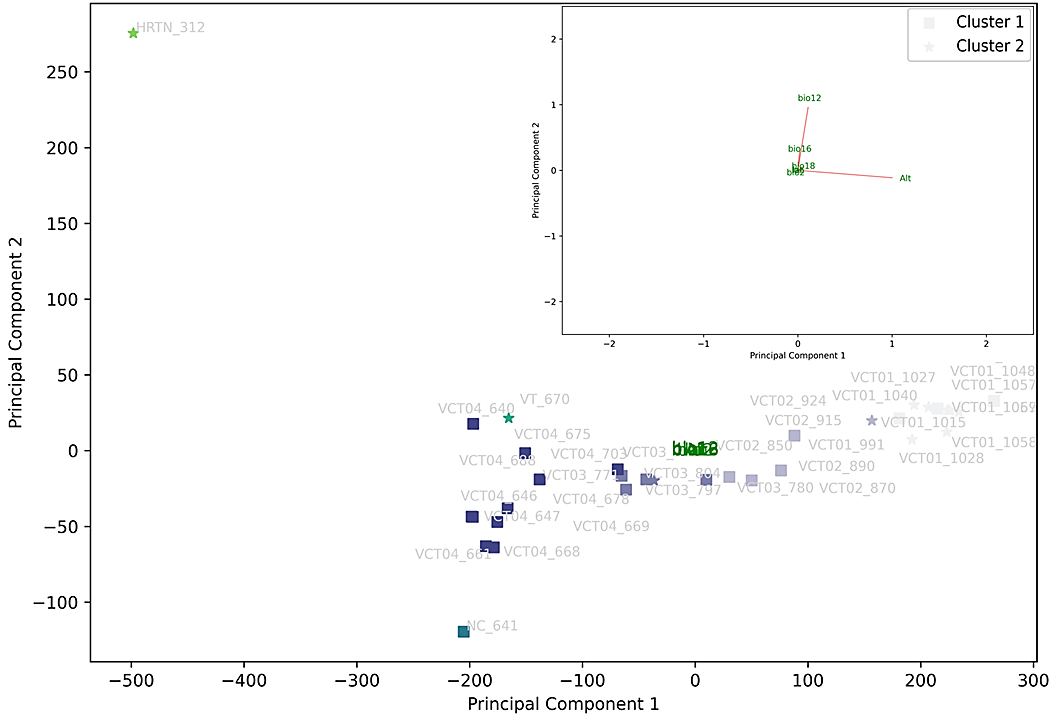


**Supplementary Figure F8:** Structure analyses at varying K levels (from top to bottom: 2, 3, 6, 9) allow detailed insights into population structure of *Diphasiastrum digitatum* genotyped using 13 microsatellites. Population subdivision is labelled at the bottom, at 7 subpopulations (VCT_01 through VCT04; NC; VT; TN_HR) and at 35 collections sites accompanied by their respective altitude above sea level [m]. Individuals are sorted per collection site by all inferred clusters’ proportions.


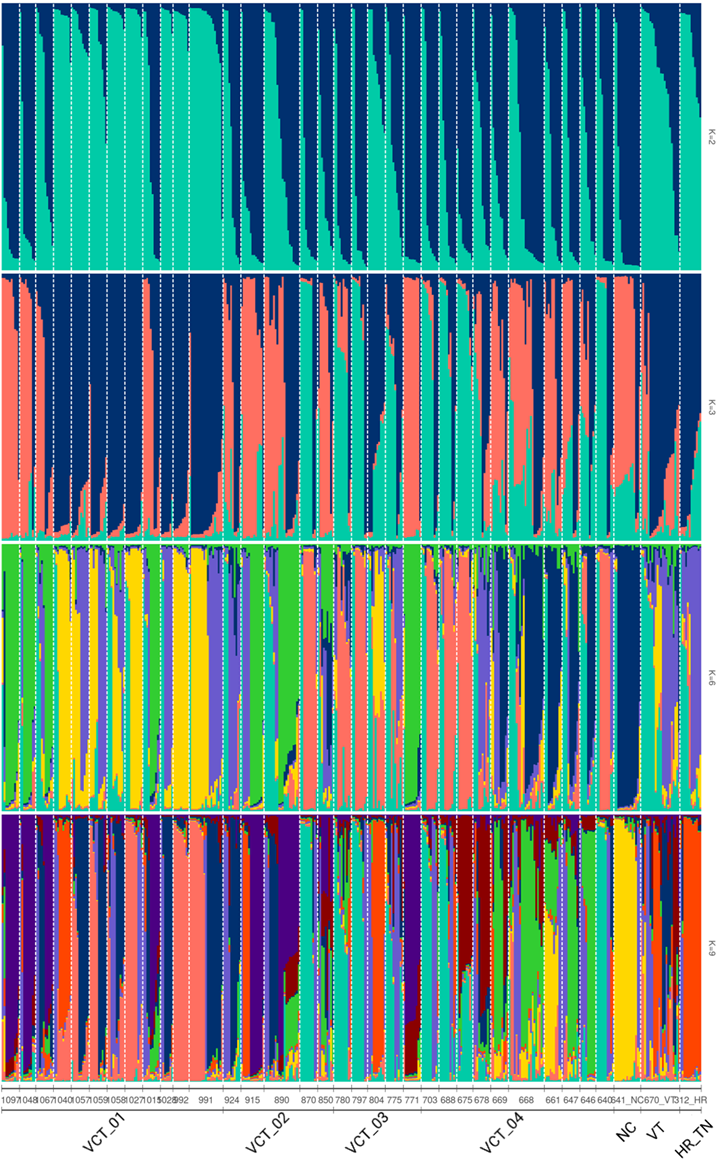


**Supplementary Figure F9:** DIYABC scenarios considered, and their respective results. For each scenario, population codes (see Table 1) are given. Estimated medians of effective population sizes are given in brackets. Estimated medians of split times (in generations) into the coalescent are presented along the right-hand time schematic. Relative direct (DR) and logistic regression (LR) support scores are respectively appended, with their 95%CI. Scenario 1 assumed TN as the starting population, from which the Virginia Creeper Trail population diverged, later giving rise sequentially to the VT and then the NC populations. Scenario 2 assumed the VT as the starting population, from which the Virginia Creeper Trail population diverged, later giving rise sequentially to the TN and then the NC populations. Scenario 3 assumed TN as the starting population, from which the Virginia Creeper Trail population diverged, later giving rise sequentially to the NC and then the VT populations. Scenario 4 assumed the NC as the starting population, from which the Virginia Creeper Trail population diverged, later giving rise sequentially to the TN and then the VT populations. Scenario 5 assumed the NC as the starting population, from which the Virginia Creeper Trail population diverged, later giving rise sequentially to the VT and then the TN populations. Scenario 6 assumed the VT as the starting population, from which the Virginia Creeper Trail population diverged, later giving rise sequentially to the NC and then the TN populations. Scenario 7 assumed the NC as the starting population, from which the Virginia Creeper Trail population diverged, later giving rise at concurrent split to the TN and the VT populations. Scenario 8 assumed the VT as the starting population, from which the Virginia Creeper Trail population diverged, later giving rise at concurrent split to the TN and the NC populations. Scenario 9 assumed TN as the starting population, from which the Virginia Creeper Trail population diverged, later giving rise at concurrent split to the NC and the VT populations. Scenario 10 assumed an unsampled starting population, from which the Virginia Creeper Trail population diverged, later giving rise to the TN population, followed by a concurrent split to the NC and the VT populations. Scenario 11 assumed an unsampled starting population, from which the Virginia Creeper Trail population diverged, later giving rise to the NC population, followed by a concurrent split to the VT and the TN populations. Scenario 12 assumed an unsampled starting population, from which the Virginia Creeper Trail population diverged, later giving rise to the VT population, followed by a concurrent split to the NC and the TN populations.
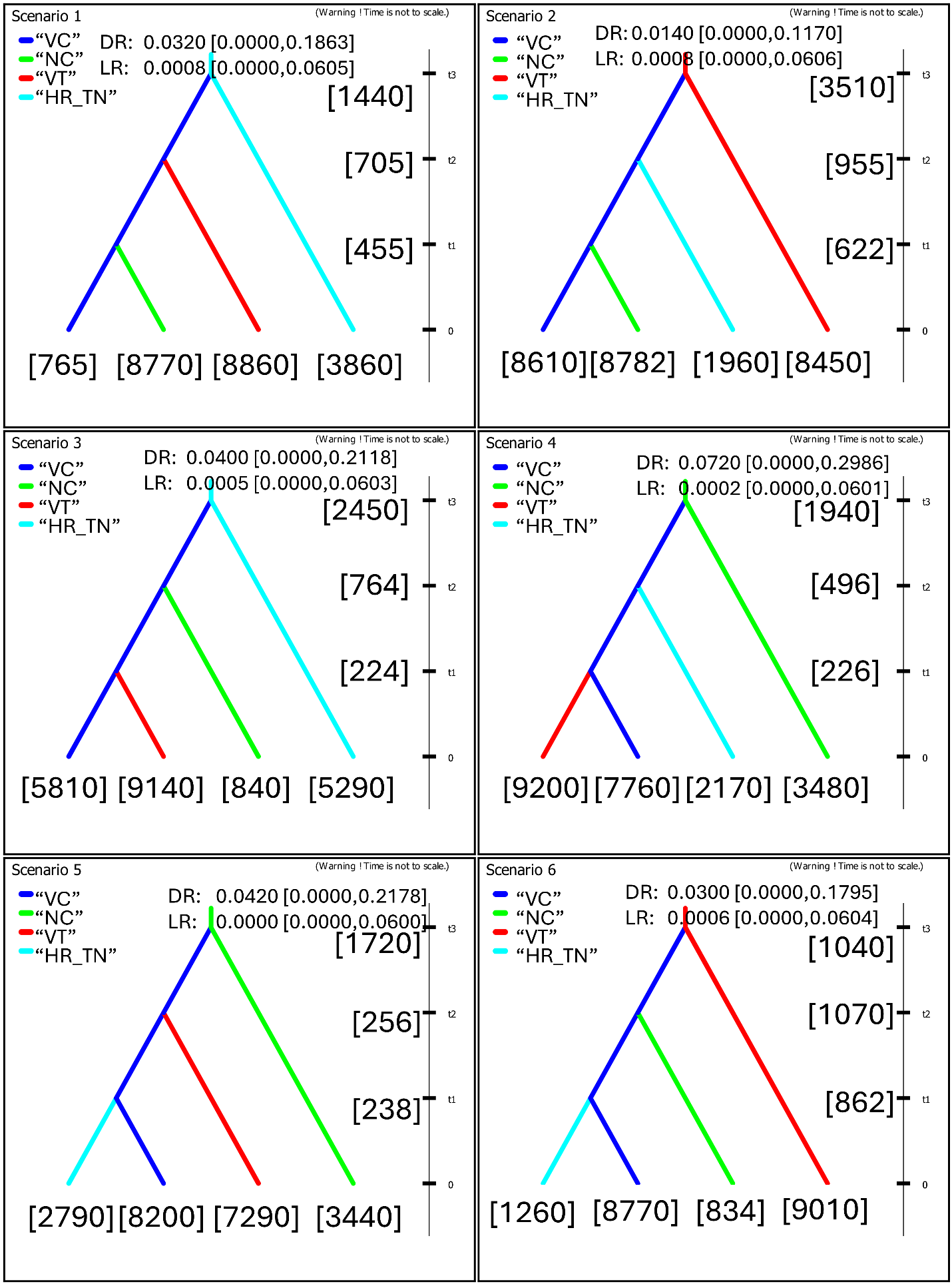

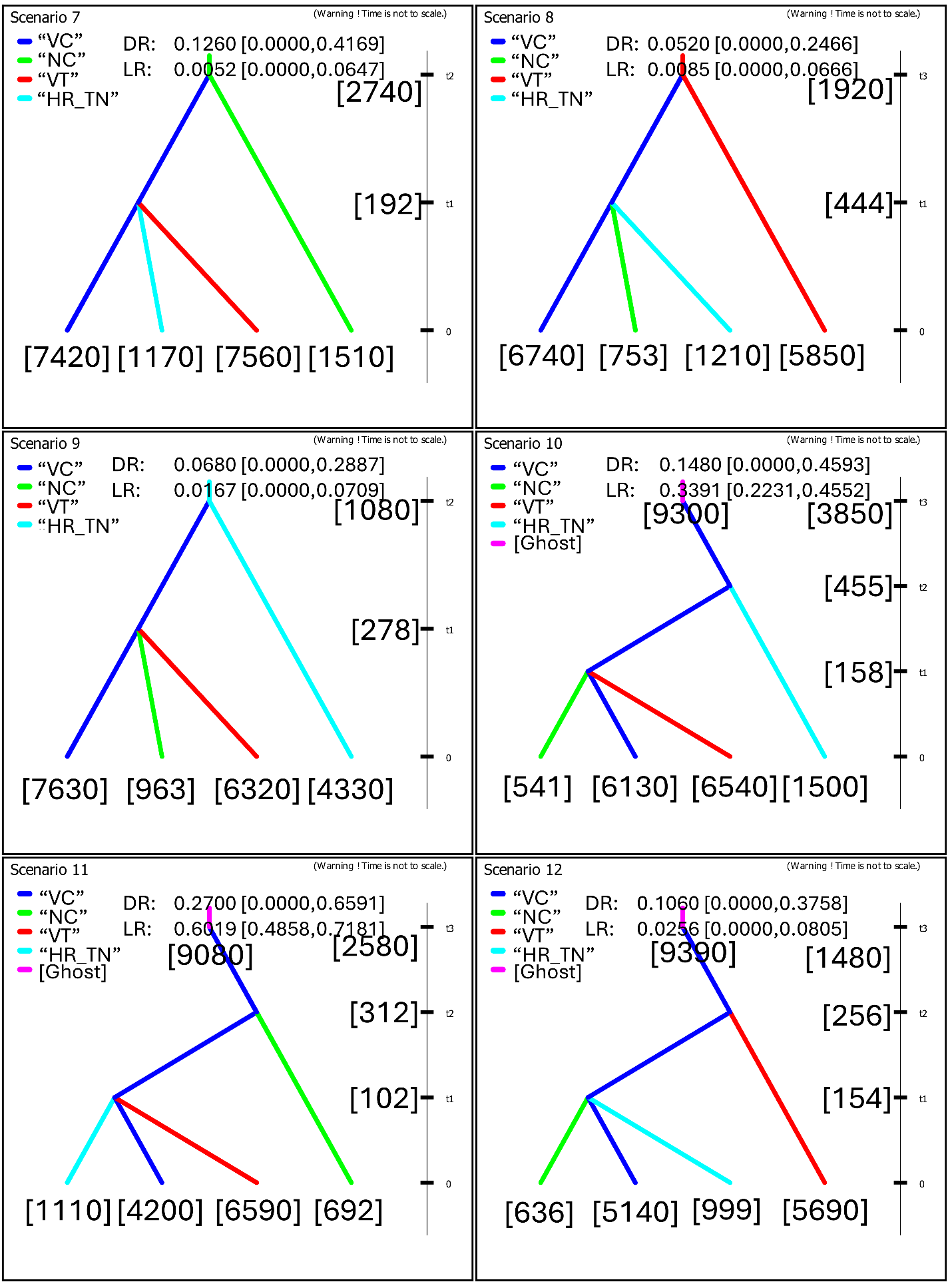


Supplementary Figure F10: Initial dataset for MaxEnt The spatial distribution of the 837 records (blue dots), relative to the extent of the training region (dark gray area); the yellow stars represent the genetic sampling sites (not used to train the models because they are outside temporal range of climate data).


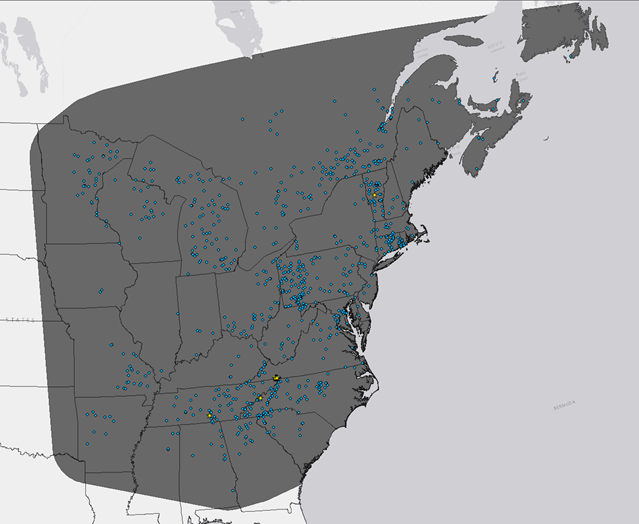

Supplement: Supplementary file 1 — Figure S1. [file ECE3-15-e71079-s002.docx]
